# Navigating the Solubility Landscape of APIs in Deep Eutectic Solvents: A Data-Driven Thermodynamic Taxonomy of Solvation Regimes and Mechanisms

**DOI:** 10.3390/molecules31091482

**Published:** 2026-04-29

**Authors:** Tomasz Jeliński, Konrad Brzózka, Maciej Przybyłek, Piotr Cysewski

**Affiliations:** 1 Department of Physical Chemistry, Faculty of Pharmacy, Collegium Medicum of Bydgoszcz, Nicolaus Copernicus University in Toruń, Kurpińskiego 5, 85-950 Bydgoszcz, Poland; 314872@stud.umk.pl (K.B.); m.przybylek@cm.umk.pl (M.P.); 2 Institute of Advanced Studies, Nicolaus Copernicus University in Toruń, Wileńska 4, 87-100 Toruń, Poland

**Keywords:** deep eutectic solvents, solubility, principal component analysis, k-means clustering, pharmaceutical formulation, data-driven design, COSMO-RS, benzocaine, lidocaine, HBA polarity

## Abstract

Deep eutectic solvents (DESs) have emerged as powerful media for enhancing the solubility of poorly water-soluble active pharmaceutical ingredients (APIs). However, their rational design remains challenging due to the complex interplay of intermolecular interactions and non-ideal thermodynamic behavior. This study develops a comprehensive, data-driven taxonomy of solute–solvent systems by integrating COSMO-RS-derived descriptors with principal component analysis (PCA) and unsupervised clustering. This approach establishes a constrained, evidence-based decision framework, which is more appropriate for complex physicochemical systems like DESs than traditional empirical rules. The analysis successfully reduces the multidimensional descriptor space to five physically interpretable axes: solvation driving force, API thermodynamic stability, solvent interaction profile, hydrogen-bond network strength, and hydration effects. Two primary solubilization mechanisms are identified: interaction-driven solvation, characterized by high API–DES affinity, and destabilization-driven solvation. Furthermore, comparison of dry and water-containing systems reveals that water acts as a thermodynamic structuring agent, fundamentally reducing system dimensionality and promoting the emergence of more distinct solvation regimes. Validated through the projection of benzocaine and lidocaine, this framework enables a transition from trial-and-error screening to mechanism-guided formulation design, providing a robust roadmap for navigating the complex solubility landscape of pharmaceutical DESs.

## 1. Introduction

Poor aqueous solubility remains one of the key challenges in modern pharmaceutical development. It is estimated that around 40% of marketed drugs and up to 90% of compounds currently under development fall into Classes II and IV of the Biopharmaceutics Classification System (BCS) [1,2,3]. These substances are characterized by low solubility and often low permeability, which leads to unpredictable absorption, reduced bioavailability, and the need for higher doses, thus increasing the risk of side effects and therapeutic failure [4,5]. For this reason, developing effective and sustainable strategies to improve solubility has become a major focus in pharmaceutical research, linking fundamental science with real clinical needs [6,7,8].

Traditional methods for enhancing solubility, such as organic co-solvents, surfactants, cocrystals, the use of salts, or complexing agents like cyclodextrins, are, of course, widely used [9,10,11]. However, they have their limitations; e.g., organic solvents can pose toxicity and environmental concerns, while some surfactants may cause irritation in patients [12,13]. In recent years, deep eutectic solvents (DESs) have emerged as a promising alternative [14,15,16]. These systems are typically formed by combining a hydrogen bond acceptor (HBA) with a hydrogen bond donor (HBD), resulting in a mixture with a significantly lower melting point than its individual components [17,18]. DESs are often referred to as “designer solvents” because their properties can be easily tuned [19,20,21]. They are also attractive due to their low toxicity, biodegradability, and relatively simple preparation from readily available components such as choline chloride, organic acids, or sugars [22,23,24].

Despite these advantages, rational design of DES-based formulations is still challenging. The solubility of an API in a DES cannot be predicted by simply looking at the individual components. Instead, it depends on a complex network of intermolecular interactions, including hydrogen bonding, van der Waals forces, electrostatic interactions, and π–π stacking [25,26,27,28,29]. Additionally, these interactions take place in a structured and often highly viscous environment [30,31]. As a result, selecting an appropriate DES has largely relied on trial-and-error approaches. While high-throughput screening can identify effective systems, it does not provide much insight into the underlying thermodynamic mechanisms [32,33,34]. This lack of understanding makes it difficult to develop general rules for solvent selection.

To address this issue, researchers increasingly turn to computational methods. One of the most useful tools in this area is the Conductor-like Screening Model for Real Solvents (COSMO-RS), which enables prediction of thermodynamic properties in complex mixtures [35,36,37]. Unlike simpler methods, COSMO-RS is based on quantum chemical calculations and describes molecules through their surface charge density distributions [38,39,40]. This makes it possible to estimate chemical potentials and activity coefficients and ultimately link molecular structure with macroscopic properties such as solubility. However, the large number of descriptors generated by COSMO-RS can make interpretation difficult [41,42,43].

This is where data-driven approaches, and more specifically machine learning (ML), become particularly valuable. In recent years, the integration of ML into pharmaceutical sciences has revolutionized the way researchers handle high-dimensional chemical data, offering powerful tools to uncover hidden patterns and complex relationships that traditional statistical methods might overlook [44,45]. Within this scope, unsupervised learning algorithms are uniquely suited for exploratory analysis, as they allow for identifying the intrinsic structure of a dataset without the need for prior labeling [46,47]. Techniques such as Principal Component Analysis (PCA) allow for reducing the complexity of the data while preserving the most important physicochemical information [48,49,50]. When combined with clustering methods like K-means [51], it becomes possible to group systems into distinct “solvation regimes” with similar underlying behavior. Rather than offering a simple predictive rule, this machine learning-enhanced approach provides a structured, evidence-based framework that better reflects the complexity of DES systems [29,52,53,54].

An important aspect that is often underestimated in DES research is the role of water. Due to the hygroscopic nature of many DES components, the presence of water is almost unavoidable in practice. Traditionally, water has been treated as either an impurity or a viscosity-reducing additive. However, recent studies show that water plays a much more active role by modifying the internal structure of the solvent. It can significantly alter the hydrogen-bond network and change the overall solvation environment [55,56,57]. Understanding how water influences the balance between API–DES and API-water interactions is crucial, especially since pharmaceutical formulations are likely to encounter aqueous environments in the body or during storage [25,58,59,60].

In this work, we address these challenges by developing a data-driven classification of API solubility in both dry and hydrated DES systems. Using a large set of COSMO-RS descriptors, we apply PCA to identify five key factors that govern solubility: the driving force of solvation (API-DES affinity), API thermodynamic stability, solvent interaction profile, hydrogen-bond network strength, and the effect of hydration. We further validate this framework using clustering methods and show that the presence of water reduces the complexity of the system, leading to more clearly defined solvation regimes. A key contribution of this study is shifting the focus from prediction to understanding mechanisms. We identify two main solubilization pathways: interaction-driven solvation, where strong API-DES interactions dominate, and destabilization-driven solvation, where increased solubility results from reduced stability of the API in the solvent phase. To demonstrate the practical relevance of this approach, we analyze the behavior of two model compounds, i.e., benzocaine and lidocaine, both widely used local anesthetics frequently employed as model poorly water-soluble drugs [61], showing how their solubility depends on both the DES composition and the level of hydration. It is important to mention that the solubility of lidocaine was studied in DESs previously. Palmelund et al. determined the solubility of this API in some eutectic systems and utilized COSMO-RS for solubility prediction [62]. A similar approach was adopted by Santiago et al., who found that a DES based on proline and lactic acid offers a substantial increase in lidocaine solubility [63]. Finally, Padilla et al. studied the dissolution behavior of lidocaine in some selected hydrophobic DES [64].

Overall, this work provides a more physically grounded perspective on the behavior of DES systems. By capturing the interplay between solute properties, solvent structure, and the influence of water, we move towards a more rational, mechanism-based approach to formulation design. The proposed framework can serve as a guide for navigating the complex chemical space of deep eutectic solvents and support the development of more effective drug delivery systems for poorly soluble compounds.

## 2. Results and Discussion

### 2.1. Solubility of Lidocaine and Benzocaine in Polar and Non-Polar DES

The experimental solubility of two active pharmaceutical ingredients (APIs), namely lidocaine and benzocaine, was evaluated across a diverse range of deep eutectic solvents (DESs), comprising such hydrogen bond acceptors (HBAs) as menthol (Men) and choline chloride (ChCl), as well as a number of polyols as hydrogen bond donors (HBDs), including tetraethylene glycol (TRG), triethylene glycol (TEG), diethylene glycol (DEG), ethylene glycol (ETG), 1,2-propanediol (P2D), and 1,3-butanediol (B3D). All solubilities were measured at 25 °C. The results, expressed in both mass concentration (mg/mL) and mole fraction, reveal significant variations in solubilization capacity depending on the chemical nature of the HBAs, the structure of the HBDs, and their molar ratio.

The most prominent observation is the dramatic difference in solubility between polar, ionic DES (ChCl-based) and hydrophobic, non-polar DES (menthol-based). For both APIs, menthol-based systems proved to be far superior solubilizers. In the case of lidocaine, the highest solubility was recorded in the Men-B3D 1:3 system at 327.21 mg/mL, which was significantly higher than the solubility in the corresponding ChCl-B3D 1:3 system (68.48 mg/mL). A similar trend was observed for benzocaine, which reached 137.56 mg/mL in Men:B3D 1:3 compared to 63.65 mg/mL in ChCl:B3D 1:3. These findings suggest that hydrophobic interactions and the specific spatial structure of menthol provide a more favorable environment for stabilizing these APIs than the ionic network of choline chloride.

Among the tested donors, 1,3-butanediol (B3D) consistently yielded the highest mass concentrations (mg/mL). This performance likely stems from an optimal combination of aliphatic chain length and the positioning of hydroxyl groups, which facilitates the formation of a robust hydrogen-bonded network with the API molecules. However, the analysis of mole fraction revealed a distinct thermodynamic preference. While for lidocaine the Men:B3D 1:3 system remained the most effective in both mass concentration (327.21 mg/mL) and mole fraction (x = 0.1844), in the case of benzocaine the observations were different. While Men:B3D was the leader in terms of mass (137.56 mg/mL), the highest mole fraction was observed for the tetraethylene glycol (TRG) system in the Men:TRG 1:3 mixture (x = 0.1292). This discrepancy is due to the higher molecular weight of TRG: although it can dissolve fewer grams of API per milliliter, the molar ratio of API to solvent molecules is the most favorable in this system.

Furthermore, within the glycol series, a clear trend was observed where solubility increased with the length of the glycol chain (TRG > TEG > DEG > ETG). This confirms that an increase in the number of ethoxy units enhances the solubilization of these specific APIs.

**Figure 1:**
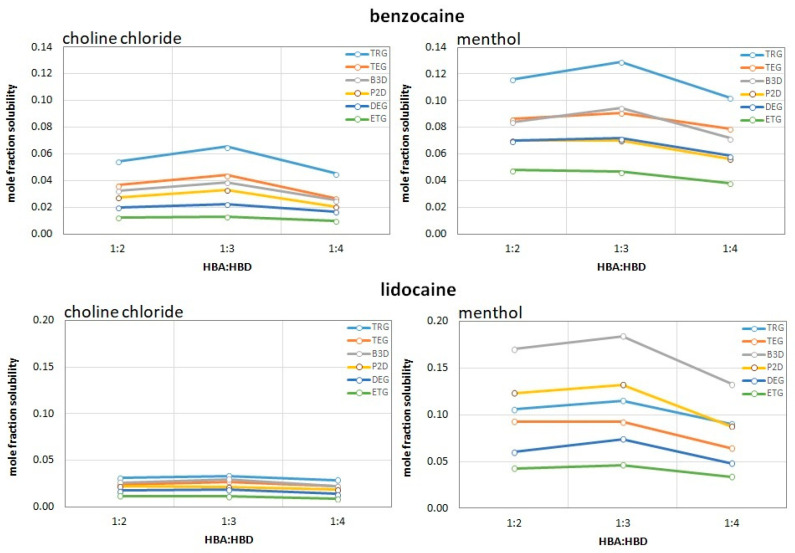
Mole fraction solubilities of benzocaine and lidocaine in the studied DES systems.

The HBA:HBD molar ratio played a critical role in determining solubility. In the vast majority of cases, the 1:3 ratio was identified as optimal. Increasing the HBD content to 1:4 almost universally led to a decrease in solubility, likely due to the dilution of the active HBA sites or unfavorable changes in the solvent’s viscosity and structural integrity. Conversely, the 1:2 ratio often appeared insufficient to fully engage the API in the eutectic network. The results of the performed experiments are collected in Figure 1.

To further investigate the role of water in DES formulations, the solubility of lidocaine and benzocaine was measured in aqueous mixtures of the most effective choline chloride-based DES. ChCl:B3D 1:3 was selected for lidocaine and ChCl:TRG 1:3 for benzocaine, as these systems exhibited the highest mass concentrations in their anhydrous states among all studied polar DES. The mole fraction of water (x_w_) in the system varied from 0.0 to 0.9 at 0.1 intervals.

The results reveal a distinct, non-linear solubility profile characterized by a co-solvency effect at low water concentrations. For both APIs, the maximum solubility expressed in mg/mL was observed at x_w_ = 0.1. Specifically, lidocaine solubility increased from 68.48 mg/mL to 78.49 mg/mL, while benzocaine solubility rose from 67.83 mg/mL to 71.87 mg/mL. This enhancement can be likely attributed to the well-documented reduction in viscosity of hydrated ChCl-polyol DESs. Analogous ChCl–glycol DESs exhibit high viscosities in the anhydrous state (typically 100–500 mPa·s at 25 °C), which decrease markedly even with small water additions [65,66,67].

However, as the water content increases beyond x_w_ = 0.1, a rapid and monotonic decline in solubility is observed for both compounds. At x_w_ = 0.5, the solubility of lidocaine drops to 28.34 mg/mL (a roughly 60% decrease from the peak), and benzocaine drops even more significantly to 16.24 mg/mL (a roughly 77% decrease). In the highly aqueous regime (x_w_ = 0.9), the solubility values reach their minimum, reflecting the poor aqueous solubility of these hydrophobic APIs.

Interestingly, the mole fraction solubility shows slightly different behavior. While for lidocaine the mole fraction also peaks at x_w_ = 0.1 (x = 0.0317), for benzocaine, the mole fraction begins to decrease immediately even at the lowest hydration level (x = 0.0655 at x_w_ = 0.0 versus x = 0.0637 at x_w_ = 0.1).

These observations show that at low concentrations, water refines the solvation environment, but at higher concentrations, it triggers a transition toward an aqueous-like regime where the anti-solvent effect dominates. This sharp decline in solubility emphasizes the need for a precise understanding of hydration levels when designing DES-based pharmaceutical formulations, as even moderate moisture uptake can significantly compromise the solvent’s loading capacity.

The distinction between mass-based solubility (mg/mL) and mole-fraction solubility (x) provides important thermodynamic insight. While mass-based concentration reflects the practical loading capacity of the formulation, the mole-fraction scale is directly connected to the thermodynamic activity of the API in the solvent phase. Consequently, changes in the mole fraction more faithfully capture variations in the chemical potential and the non-ideal mixing behavior that govern the true driving force for dissolution.

Upon water addition, two opposing effects occur simultaneously. First, even small amounts of water (x_w_ = 0.1) significantly decrease the average molecular weight of the DES mixture, which artificially increases the numerical value of the mole fraction for a given mass of dissolved API. Second, water modifies the intermolecular interactions and lowers the viscosity, thereby altering both the activity coefficient and the overall solvation environment. These solvent composition effects explain why the mass-based and mole-fraction solubility profiles diverge slightly between lidocaine and benzocaine. The link between these macroscopic observations and the microscopic thermodynamic descriptors obtained from COSMO-RS is explicitly captured in the PCA presented in Section 2.2, Section 2.3, Section 2.4 and Section 2.5, offering a consistent, activity-based framework for interpreting hydration-induced changes in DES systems.

Detailed solubility values obtained for lidocaine and benzocaine in the studied systems can be found in the Supplementary Materials (see Section S1).

### 2.2. Dimensionality Reduction and Intrinsic Complexity of DES Systems

**Figure 2:**
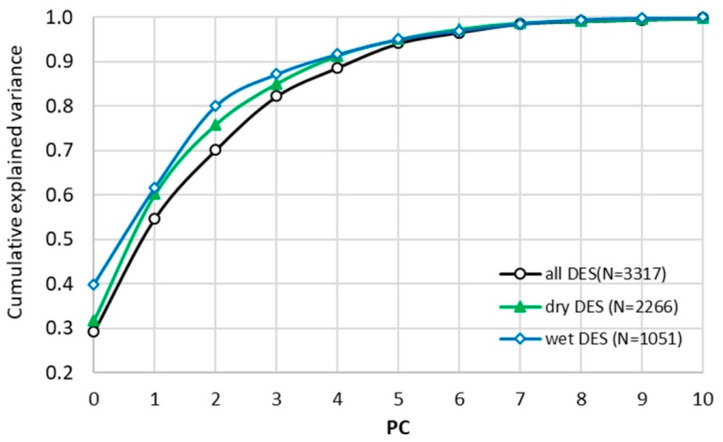
Results of dimensionality reduction for the full dataset and the two subsets comprising water-containing and water-free API-DES systems.

Principal component analysis (PCA) was employed to reduce the dimensionality of the descriptor space derived from COSMO-RS calculations while preserving the physicochemical information relevant to solubility. The cumulative explained variance analysis revealed that four principal components (PCs) are sufficient to capture the majority of the variance across all datasets, with values exceeding 82%, 85%, and 87% for the full, dry, and wet datasets, respectively. Inclusion of a fifth component increased the explained variance above 90%, confirming that the descriptor space is moderately low-dimensional despite its apparent complexity. The results can be seen in Figure 2.

A notable distinction was observed between dry and water-containing systems. Wet DES systems reached approximately 80% explained variance already at three principal components, whereas dry systems required four components to achieve comparable coverage. This indicates that the presence of water reduces the intrinsic dimensionality of the system. Such behavior suggests that water acts as a structuring agent, imposing dominant intermolecular interactions that effectively constrain the degrees of freedom governing solubility. The retention of five PCs was guided by both the cumulative explained variance (>90%) and the clear physical interpretability of PC5 as the hydration-competition axis, essential for distinguishing dry and wet systems.

The PCA loadings exhibited high persistence across all datasets, confirming that the extracted latent structure is robust and not an artifact of dataset composition. Minor variations in loadings between dry and wet systems reflect physically meaningful modulation of intermolecular interactions rather than statistical instability. A detailed loading persistence analysis is provided in the Supplementary Materials (see Section S2). All subsequent multivariate analyses, including K-means clustering and system projections, were performed in the five-dimensional PCA space defined by the first five principal components.

### 2.3. Physicochemical Interpretation of Principal Components

The interpretation of PCA loadings enables direct assignment of physicochemical meaning to the latent variables and elucidation of the fundamental factors governing API solubility in DES systems. The high consistency of loadings across the full dataset, as well as dry and wet datasets, confirms that the extracted latent variables represent intrinsic physicochemical relationships embedded in the COSMO-RS descriptor space rather than dataset-specific artifacts (see Section S2).

**Figure 3:**
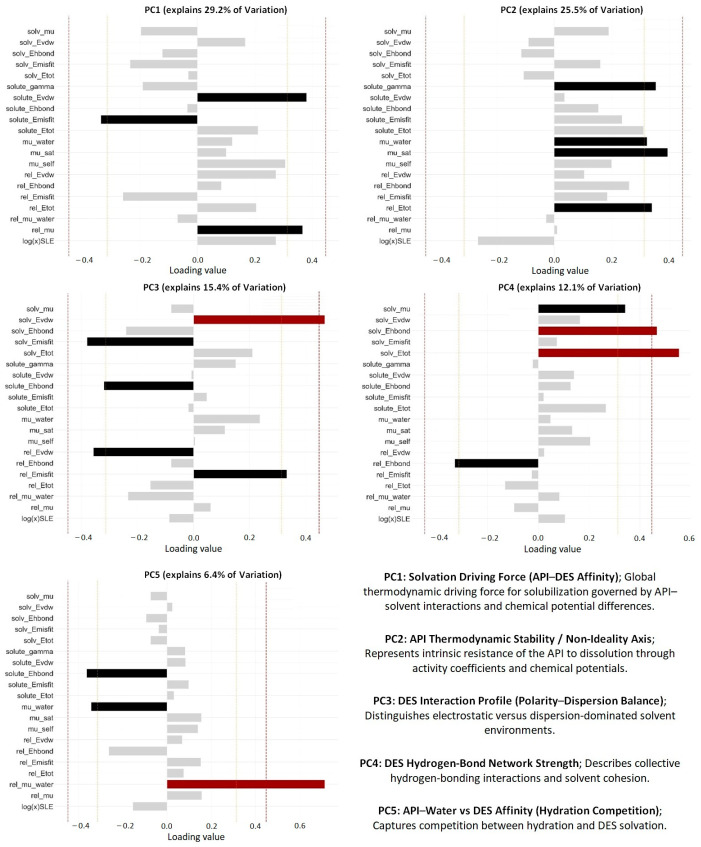
Contributions of COSMO-RS-derived descriptors to the orthogonal principal components and intrinsic physicochemical relationships embedded in the descriptor space related to each principal component of the whole dataset of API solubility in DES. Contributions highlighted in black denote descriptors with moderate absolute loadings (≥0.31, light vertical line), while those highlighted in red denote descriptors with significant absolute loadings (≥0.45, dark vertical line).

Each principal component can therefore be associated with a distinct thermodynamic aspect governing solubility in deep eutectic solvents (DES). The contributions of COSMO-RS-derived descriptors to the orthogonal principal components are summarized in Figure 3.

The first principal component (PC1, 20.8% variance) is defined as the solvation driving force (API–DES affinity). It captures the global thermodynamic balance between favorable API–DES interactions and energetic penalties associated with intermolecular mismatch. Positive loadings are dominated by solute_Evdw (0.38, moderate) and rel_mu (0.37, moderate), indicating that favorable dispersion interactions and the chemical potential gradient between API and DES promote solubilization. The negative contribution of solute_Emisfit (−0.34, moderate) reflects the opposing effect of electrostatic mismatch, consistent with reduced compatibility between polar environments. The presence of log(x)SLE (0.27) within this component confirms its direct connection to experimental solubility, establishing PC1 as the primary axis governing dissolution behavior.

The second principal component (PC2, 17.3% variance) represents the API thermodynamic stability and non-ideality axis. It is characterized by moderate positive contributions from mu_sat (0.40), solute_gamma (0.35), mu_water (0.32), and solute_Etot (0.31), which collectively describe the thermodynamic barrier opposing solubilization. The contribution of rel_Etot (0.34) further highlights the role of energy differences between API and DES phases in determining non-ideal behavior. This component reflects the intrinsic resistance of the API to dissolution: high PC2 values correspond to thermodynamically stable APIs with high activity coefficients, while low PC2 values indicate destabilization and reduced energetic barriers to dissolution.

The third principal component (PC3, 13.9% variance) captures the DES interaction profile (polarity–dispersion balance). The strong positive loading of solv_Evdw (0.47, strong) represents cohesive dispersion interactions within the DES, while negative contributions from solv_Emisfit (−0.38, moderate) and solute_Ehbond (−0.32) reflect opposing electrostatic and hydrogen-bonding effects. The contrasting signs of rel_Evdw (−0.36) and rel_Emisfit (0.33) confirm that this axis differentiates dispersion-dominated from polarity-dominated solvent environments. This distinction is critical for understanding how solvent composition governs the dominant intermolecular interaction mechanisms.

The fourth principal component (PC4, 11.5% variance) represents the DES hydrogen-bond network strength. It is dominated by strong positive loadings from solv_Etot (0.55) and solv_Ehbond (0.47), which directly reflect the total cohesive energy and hydrogen-bonding contribution within the DES. The moderate contribution of solv_mu (0.34) indicates that this structural cohesion influences the chemical potential of the solvent, while the negative loading of rel_Ehbond (−0.33) suggests that the relative hydrogen-bonding balance between API and DES modulates solubility outcomes. High PC4 values therefore correspond to strongly interconnected hydrogen-bond networks that define solvent structure and influence solute accommodation.

The fifth principal component (PC5, 8.9% variance) captures the competition between API hydration and DES solvation. This component is dominated by rel_mu_water (0.71, strong), which quantifies the chemical potential difference in the API between saturated and aqueous states. Negative contributions from solute_Ehbond (−0.36) and mu_water (−0.34) indicate that API hydrogen-bonding propensity and affinity for water counterbalance this effect. PC5 therefore reflects the fundamental trade-off between hydration and DES affinity, providing insight into how water modulates solubility behavior. Although this component contributes less to overall variance, it becomes particularly relevant when considering systems with varying water content.

Importantly, comparison of loading structures between dry and all data-containing subsets (see Supplementary Section S3) reveals systematic differences that provide additional mechanistic insight. In dry DES systems, the dominant contributions to PC1 and PC2 are more strongly associated with electrostatic (misfit) and activity-related descriptors, indicating that solubility is governed by relatively independent contributions from different interaction types. This leads to a more decoupled descriptor space, consistent with the higher effective dimensionality observed for dry systems.

In contrast, wet DES systems exhibit a redistribution of loadings, where chemical potential, solvent interaction energies, and hydration-related descriptors contribute more coherently within fewer principal components. In particular, the increased influence of water-related terms across multiple components reflects a coupling of thermodynamic contributions, effectively compressing the descriptor space. This behavior indicates that water acts as a dominant structuring agent, simultaneously influencing API stability, solvent energetics, and interaction balance.

The PCA results demonstrate that the complex multidimensional descriptor space can be reduced to a small number of physically interpretable axes corresponding to distinct thermodynamic mechanisms. The observed differences between dry and wet systems further highlight the role of water in modulating and coupling these mechanisms, providing a coherent framework for understanding solubility in DES systems.

### 2.4. Cluster Validation and Emergence of Thermodynamic Regimes

Clustering of the reduced descriptor space was performed using a structured validation protocol designed to ensure both statistical robustness and physical interpretability of the resulting partitions. The procedure, referred to here as the protocol validation study, evaluates clustering solutions across a wide range of cluster numbers (k), explicitly extending beyond visually apparent “elbow” points in order to avoid premature or biased selection of the optimal partition.

The clustering workflow employs a custom initialization strategy (NANI, Nearest-Average Non-Iterative initialization), which combines medoid-based selection with a farthest-point heuristic analogous to k-means++ to ensure stable and well-separated initial centroids. As implemented, the procedure first identifies a global medoid minimizing total pairwise distances, followed by iterative selection of maximally distant points to span the descriptor space. This approach improves reproducibility and reduces sensitivity to random initialization.

For each value of k, clustering quality was evaluated using two complementary metrics: (i) the minimum Mahalanobis distance between clusters, which quantifies statistical separation in multivariate space, and (ii) the silhouette coefficient, which reflects the balance between intra-cluster cohesion and inter-cluster separation. A threshold criterion of Mahalanobis distance > 3.0 was applied to ensure that only statistically meaningful partitions were considered acceptable. The Mahalanobis distance threshold of 3.0 was adopted as a conservative criterion for statistical separation, corresponding to approximately three standard deviations in multivariate space and consistent with established practices in PCA-based clustering [68].

**Figure 4:**
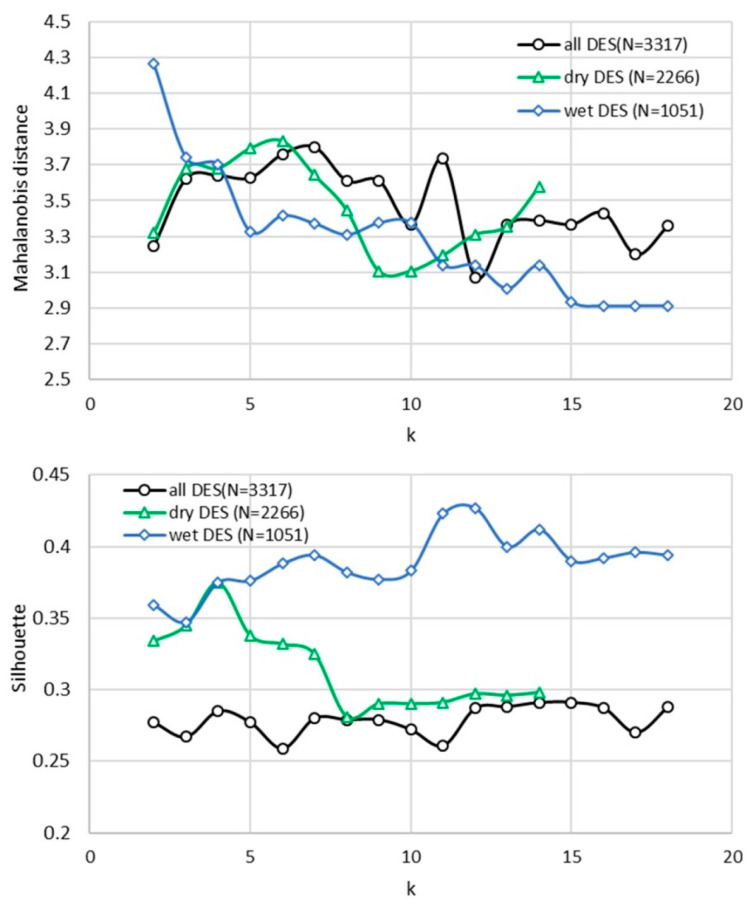
Validation of clustering solutions as a function of the number of clusters (k) for the full dataset (all DES), dry DES, and wet DES systems. Clustering quality is assessed using the minimum Mahalanobis distance between clusters (measure of statistical separation) and the silhouette coefficient (measure of intra-cluster cohesion and inter-cluster separation). The dashed horizontal line indicates the acceptance threshold for Mahalanobis distance (3.0), used to identify statistically meaningful partitions.

Figure 4 presents the dependence of both metrics on the number of clusters for the full dataset as well as the dry and wet subsets. Several important observations emerge from this analysis.

For the full dataset, both Mahalanobis distance and silhouette values remain relatively stable across a broad range of cluster numbers (k = 2–18), with all tested configurations satisfying the separation criterion. The silhouette coefficient varies only modestly (≈0.26–0.29), and no pronounced maximum is observed. This behavior indicates the absence of a single optimal partition and suggests that the descriptor space forms a quasi-continuous thermodynamic manifold rather than a set of sharply separated groups.

A similar trend is observed for dry DES systems, although a shallow maximum in silhouette appears at k = 4 (≈0.375), indicating that a limited number of clusters can capture the dominant variability. Nevertheless, the overall weak dependence of clustering metrics on k confirms that the underlying space remains largely continuous. This observation is consistent with the PCA, which showed that electrostatic, dispersion, and hydrogen-bonding contributions remain relatively decoupled in dry systems, resulting in a broadly distributed and overlapping descriptor space.

In contrast, wet DES systems exhibit a markedly different behavior. The silhouette coefficient increases systematically with increasing k, reaching values above 0.42 for k = 11–12, while maintaining acceptable Mahalanobis separation up to k ≈ 14. Beyond this range, the Mahalanobis distance falls below the acceptance threshold, indicating over-partitioning and loss of statistical distinctness. The presence of a clear maximum in clustering quality metrics demonstrates that wet systems exhibit well-defined clustering structure, consistent with the emergence of discrete thermodynamic regimes.

Despite the application of quantitative validation metrics, the selection of the optimal number of clusters (k) remains inherently non-unique, particularly for the full and dry datasets. Rather than enforcing a single optimal solution, the applied protocol defines a mathematically constrained solution space, within which clustering results satisfy minimum statistical separation criteria. The combined use of Mahalanobis distance and silhouette coefficient reduces arbitrariness by excluding statistically invalid partitions, even though it does not impose a unique optimum. The clustering structure remains consistent when restricted to lower-dimensional subspaces (PC1–PC2), supporting the robustness of the five-PC solution. This observation is consistent with the PCA, which showed that electrostatic, dispersion, and hydrogen-bonding contributions remain relatively decoupled in dry systems, resulting in a broadly distributed and overlapping descriptor space.

Importantly, this behavior reflects the physicochemical nature of DES systems rather than a limitation of the methodology. In systems governed by continuous variations in intermolecular interactions, clustering serves as a discretization of an underlying thermodynamic continuum rather than identification of inherently discrete classes. Consequently, the selected number of clusters represents a balance between resolution and interpretability, guided jointly by statistical validation and physicochemical reasoning. The situation differs significantly in the presence of water. The emergence of well-defined optima in clustering metrics for wet systems indicates that water induces a higher degree of organization in the descriptor space, effectively coupling previously independent thermodynamic contributions. As a result, clustering in wet-DES becomes more data-driven and less ambiguous, reflecting the formation of distinct solvation regimes. Based on these considerations, representative cluster numbers were selected to balance interpretability and statistical validity. For the full dataset, a five-cluster solution was adopted as a compromise within a continuous landscape. For dry systems, four clusters were sufficient to capture the dominant variability, while for wet systems, higher-resolution partitioning (k ≈ 10–12) is justified by the presence of well-defined maxima in clustering quality metrics. The validation study demonstrates that clustering in DES systems should be interpreted as a physically informed discretization of a continuous thermodynamic space, with water playing a key role in promoting the emergence of distinct and statistically robust solvation regimes.

### 2.5. Data-Driven Taxonomy of Solute–Solvent Systems

**Figure 5:**
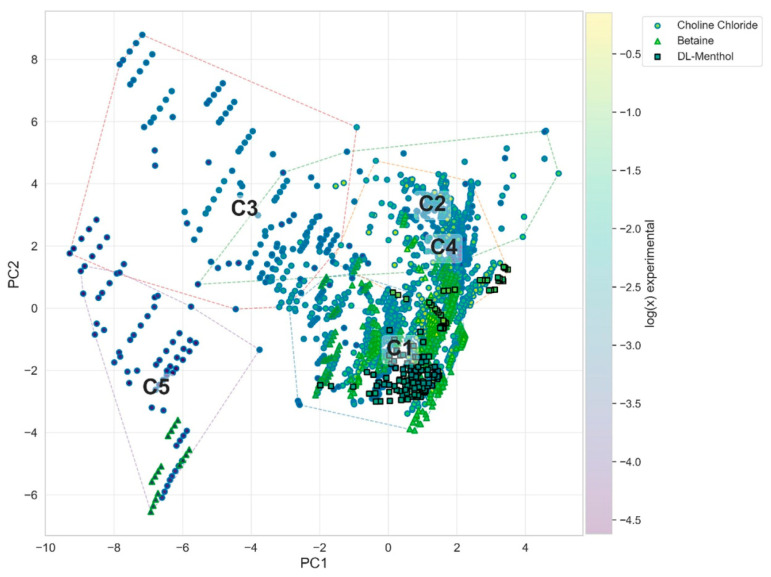
Projection of all DES systems onto the PC1–PC2 plane, representing the solvation driving force (PC1) and API thermodynamic stability/non-ideality (PC2). Points are colored according to experimental solubility (log(x)), and symbols indicate the type of hydrogen bond acceptor (HBA). Convex hulls denote the five identified clusters (C1–C5). A clear solubility gradient is observed along PC1, confirming its dominant role in governing dissolution. Clustering reveals distinct thermodynamic regimes, including destabilization-driven (C1) and interaction-driven (C4) solvation pathways, as well as regions of limited solubility (C2, C3, and C5).

Projection of all systems onto the PC1–PC2 space combined with clustering in the multidimensional PCA domain, as shown in Figure 5, reveals that these clusters represent physically meaningful regions within the solubility landscape rather than arbitrary statistical groupings, as supported by the validation protocol and the consistency of cluster separation across higher-order principal component projections (see Supplementary Section S4). The organization of clusters along PC1 and PC2 provides a direct physicochemical interpretation. PC1 represents the solvation driving force governed by API–DES affinity, while PC2 reflects the thermodynamic stability and non-ideality of the API. Together, these axes define a two-dimensional solubility landscape in which systems are positioned according to both their interaction strength and their intrinsic resistance to dissolution, more effective than relying solely on API destabilization. The distinction between these two regimes is further supported by higher-dimensional projections. As shown in the PC1–PC3 and PC1–PC4 representations (Supplementary Figures S4.1 and S4.2), clusters C1 and C4 differ in their solvent interaction profiles and hydrogen-bond network characteristics. In particular, the interaction-driven regime (C4) is associated with more defined solvent interaction patterns and, in many cases, stronger or more coherent solvent structuring, whereas the destabilization-driven regime (C1) spans a broader range of solvent environments. The remaining clusters represent less favorable or limiting regimes. Cluster C2 is characterized by a high solvation driving force but simultaneously high API stability, resulting in systems where favorable solvent interactions are insufficient to overcome the thermodynamic barrier to dissolution. Cluster C3 corresponds to weakly interacting systems with moderate to high API stability, reflecting poor compatibility between solute and solvent. Cluster C5 (failure regime) is characterized by low API–DES affinity (low scores on PC1), unfavorable polarity–dispersion balance (high PC3 indicating electrostatic mismatch), and weak hydrogen-bond network cohesion (low PC4). These conditions result in high activity coefficients (elevated PC2 contributions) and poor solute accommodation, yielding low solubility values.

Additional insight is provided by the PC1–PC5 projection (Supplementary Figure S4.3), which highlights the role of hydration effects. While hydration does not define the primary clustering structure, it introduces a secondary modulation, particularly within clusters occupying similar PC1 regions. This confirms that water acts as a modifying factor rather than a primary driver in defining solvation regimes within the full dataset. The distribution of systems across clusters further reveals a strong dependence on the identity of the hydrogen bond acceptor (HBA). Choline chloride-based systems span a wide region of the descriptor space, reflecting their versatility across different thermodynamic regimes. Betaine-based systems occupy intermediate regions, while DL-menthol-based systems are preferentially located within regions associated with favorable solvation, particularly the interaction-driven regime. This behavior reflects the reduced polarity and increased dispersion character of menthol-based systems, which promotes stronger API–DES affinity. The proposed taxonomy demonstrates that DES systems cannot be adequately described by a single descriptor or simple classification scheme. Instead, solubility emerges from the interplay of multiple thermodynamic factors, including interaction strength, API stability, solvent structure, and hydration effects. The identification of distinct regimes within this multidimensional space provides a framework for rational formulation design, enabling targeted selection of solvent systems based on the dominant mechanism of solubilization. Importantly, this taxonomy should be interpreted not as a rigid classification but as a physically informed discretization of a continuous thermodynamic landscape, in which cluster boundaries represent transitions between dominant solvation mechanisms rather than sharply defined system types.

### 2.6. Role of Hydrogen Bond Acceptors and Water in Structuring Solvation Space

The distribution of systems across the identified thermodynamic regimes reveals a strong dependence on the identity of the hydrogen bond acceptor (HBA). Choline chloride-based systems span a broad region of the descriptor space, reflecting their versatility across multiple solvation regimes. Betaine-based systems occupy intermediate regions, while DL-menthol-based systems are preferentially located within regions associated with favorable solvation, particularly the interaction-driven regime (C4). This behavior reflects the reduced polarity and increased dispersion character of menthol-based systems, which promotes stronger API–DES affinity.

**Figure 6:**
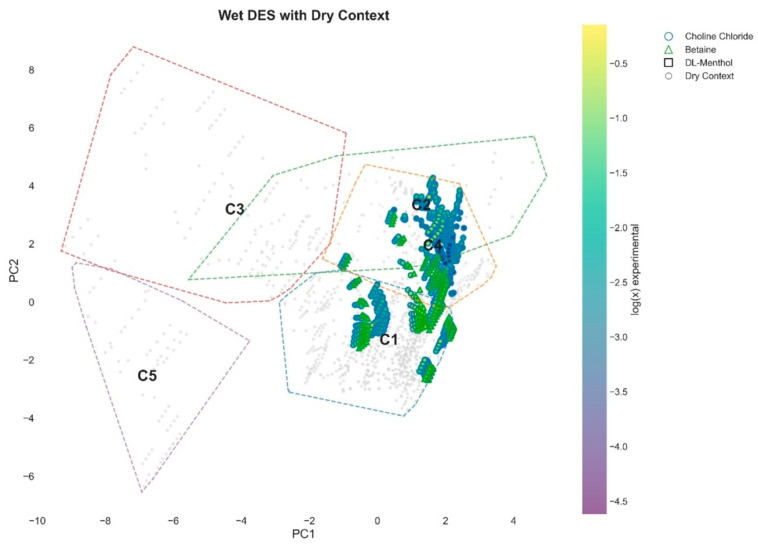
Projection of wet DES systems onto the global PCA space (PC1–PC2), shown together with the full dataset as background (grey points). Colored symbols represent wet systems, with marker type indicating hydrogen bond acceptor (HBA) and color corresponding to experimental solubility (log(x)). Cluster boundaries (C1–C5) are derived from the full dataset. Wet systems are preferentially distributed within the central and high-performance regions (C1, C2, and C4), while the failure regime (C5) remains largely unoccupied. This demonstrates that water does not introduce new solvation regimes but redistributes systems within the existing thermodynamic landscape, selectively promoting occupation of favorable regions.

While HBA identity defines the accessible regions of the thermodynamic space, additional insight is obtained by examining the effect of water on system distribution. Projection of wet DES systems onto the global PCA space in Figure 6, with the full dataset shown as background, demonstrates that water does not create new regions of solvation behavior. Instead, wet systems are redistributed within the existing thermodynamic landscape, occupying well-defined areas corresponding to specific clusters.

In particular, wet systems are predominantly located within clusters C1, C2, and C4, with a clear preference for the interaction-driven regime (C4). In contrast, the failure regime (C5) is largely unpopulated by wet systems, indicating that the presence of water suppresses unfavorable solvation conditions. This redistribution suggests that water acts as a thermodynamic filter, selectively stabilizing favorable configurations while reducing the accessibility of poorly interacting systems. These observations provide a unified interpretation of the roles of composition and hydration. While HBA identity determines the structural characteristics of the solvent and defines the potential interaction landscape, water modulates the effective thermodynamic space by promoting specific regions within that landscape. This dual effect explains the observed reduction in dimensionality and the emergence of a more distinct clustering structure in wet-DES systems.

Overall, the combined influence of HBA identity and water content demonstrates that solvation behavior in DES systems is governed not only by intrinsic interaction parameters but also by selective occupation of thermodynamically favorable regions within a continuous descriptor space.

To assess the predictive relevance of the proposed taxonomy, selected APIs (benzocaine and lidocaine) were projected onto the established PCA space without influencing the clustering procedure. This approach enables evaluation of whether the derived thermodynamic regimes correspond to meaningful and transferable solvation behavior.

**Figure 7:**
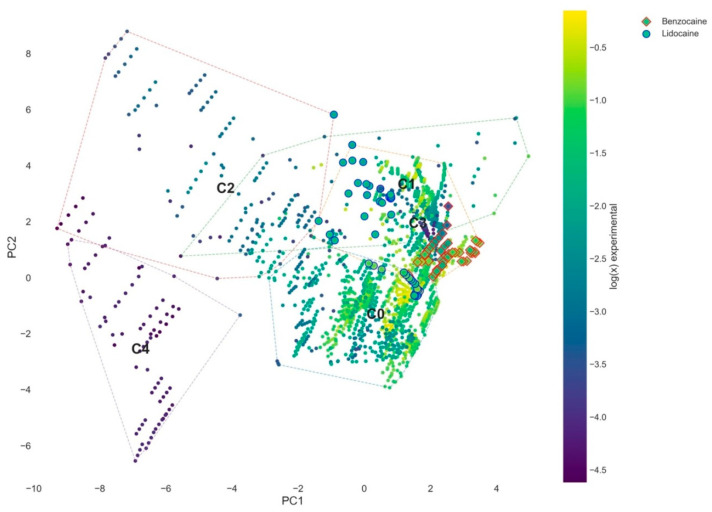
Projection of benzocaine and lidocaine systems onto the global PCA space (PC1–PC2), shown together with the full dataset as background (grey points). Colored symbols represent the selected APIs, with color corresponding to experimental solubility (log(x)). Cluster boundaries (C1–C5) are derived from the full dataset. The distinct localization of both APIs confirms that the identified clusters represent transferable and physically meaningful solvation regimes.

As shown in Figure 7, both APIs occupy well-defined regions within the PC1–PC2 space, confirming that the identified clusters represent physically meaningful solvation regimes rather than dataset-specific artifacts. The projections reveal distinct patterns for the two compounds, reflecting differences in their physicochemical characteristics and solvation mechanisms. Benzocaine systems are predominantly located in regions of high solvation driving force (high PC1), primarily within or near the interaction-driven regime (C4). These systems are associated with relatively high solubility values and exhibit a narrow distribution in the PCA space. This localization indicates that benzocaine solubility is governed primarily by favorable API–DES interactions, with limited sensitivity to variations in solvent composition. The consistency of this behavior suggests that benzocaine preferentially exploits interaction-driven solvation pathways. In contrast, lidocaine systems display a broader distribution across multiple clusters, including C1, C2, and partially C4. This dispersion reflects the presence of competing thermodynamic mechanisms, involving both interaction-driven and destabilization-driven contributions. Systems located in C1 indicate dissolution facilitated by reduced API stability, while those extending toward C2 reflect conditions in which favorable solvent interactions are insufficient to fully overcome thermodynamic resistance. The broader spread of lidocaine data is consistent with its more complex balance of intermolecular interactions and highlights its sensitivity to solvent composition. The distinct behaviors of benzocaine and lidocaine provide strong validation of the proposed taxonomy. The clustering framework successfully captures differences in solvation mechanisms between APIs and enables their classification within a unified thermodynamic space. Importantly, this demonstrates that the developed approach is not limited to describing existing data but can be used to interpret and predict API-specific solubility behavior in DES systems.

### 2.7. Implications for Pharmaceutical Formulation Design

The developed data-driven taxonomy provides a mechanistic framework for rational design of pharmaceutical formulations in deep eutectic solvents. By mapping systems onto a physically interpretable thermodynamic space, it becomes possible to identify not only favorable solvent compositions but also the underlying mechanisms governing solubility. A key outcome of this study is the identification of two distinct solubilization pathways: (i) interaction-driven solvation, characterized by strong API–DES affinity (high PC1), and (ii) destabilization-driven solvation, in which dissolution is facilitated by reduced API thermodynamic stability (low PC2). The results demonstrate that interaction-driven mechanisms, represented by cluster C4, are associated with higher solubility and more consistent performance. In contrast, destabilization-driven systems (C1) provide moderate solubility but exhibit greater variability and reduced efficiency. These findings suggest that formulation strategies should prioritize solvent systems that maximize favorable intermolecular interactions rather than relying solely on destabilization of the API. In practical terms, this involves selecting DES compositions with an appropriate balance of dispersion and polarity, as well as sufficient structural cohesion to support effective solvation. The role of hydrogen bond acceptors further supports this conclusion. DL-menthol-based systems, characterized by reduced polarity and enhanced dispersion interactions, are more likely to populate high-performance regions of the thermodynamic space. In contrast, more polar HBAs, such as choline chloride, provide broader coverage of the descriptor space but do not inherently favor optimal solvation regimes. This indicates that HBA selection can be used as a primary design parameter to target specific regions of the solubility landscape. Water plays an additional and distinct role by acting as a thermodynamic filter, redistributing systems within the existing descriptor space and promoting occupation of favorable regimes while suppressing unfavorable ones. The observed exclusion of wet systems from the failure regime (C5) and their concentration in clusters C1 and C4 demonstrate that controlled hydration can be used to enhance solubility by guiding systems toward optimal thermodynamic conditions. The projection of individual APIs further illustrates how this framework can be applied in practice. Benzocaine, which exhibits a strong tendency toward interaction-driven solvation, can be effectively formulated using solvents that enhance API–DES affinity. In contrast, lidocaine requires more careful optimization due to its sensitivity to both interaction strength and thermodynamic stability, necessitating a balanced approach that considers multiple solvation pathways.

The presented methodology enables a shift from empirical screening toward mechanism-guided formulation design, where solvent selection is informed by the position of systems within a multidimensional thermodynamic space. This approach provides a rational basis for predicting solubility behavior and optimizing DES-based formulations, with potential applicability to a broad range of pharmaceutical compounds.

The experimental validation of the proposed taxonomy was performed using two structurally related local anesthetics, i.e., benzocaine and lidocaine. While the results obtained for these two APIs are internally consistent and clearly demonstrate the applicability of the framework, we acknowledge that this limited chemical diversity constrains the breadth of the experimental validation. Consequently, the experimental part of the study primarily serves to illustrate the predictive and interpretive power of the taxonomy for individual APIs rather than to provide a comprehensive test across highly diverse chemical scaffolds. Nevertheless, the overall robustness and generality of the proposed solvation regimes are supported primarily by the large compiled dataset encompassing 23 chemically diverse APIs from multiple therapeutic classes (including NSAIDs, sulfonamides, phenolic acids, alkaloids, and others). The projection of benzocaine and lidocaine onto the established PCA space should therefore be interpreted as a successful out-of-sample validation rather than the sole basis for claiming broad applicability. Future studies extending the experimental validation to APIs with greater structural and functional diversity would further strengthen the framework.

## 3. Materials and Methods

### 3.1. Materials

The active pharmaceutical ingredients (APIs) used in this study, lidocaine (CAS: 137-58-6, Mw = 234.34 g/mol, purity ≥ 98%) and benzocaine (CAS: 94-09-7, Mw = 165.19 g/mol, purity ≥ 98%), were purchased from Sigma-Aldrich (St. Louis, MO, USA).

The deep eutectic solvents (DESs) were synthesized using various combinations of hydrogen bond acceptors (HBAs) and hydrogen bond donors (HBDs). The HBAs, also sourced from Sigma-Aldrich, included choline chloride (ChCl, CAS: 67-48-1, Mw = 139.62 g/mol, purity ≥98%) and DL-menthol (Men, CAS: 89-78-1, Mw = 156.27 g/mol, purity ≥ 99%). The following polyols were employed as HBDs: tetraethylene glycol (TRG, CAS: 112-60-7, purity ≥ 99%), triethylene glycol (TEG, CAS: 112-27-6, purity ≥ 99%), diethylene glycol (DEG, CAS: 111-46-6, purity ≥ 99%), ethylene glycol (ETG, CAS: 107-21-1, purity ≥ 99.5%), 1,2-propanediol (P2D, CAS: 57-55-6, purity ≥ 99.5%), and 1,3-butanediol (B3D, CAS: 107-88-0, purity ≥ 99%), all similarly provided by Sigma-Aldrich.

Analytical grade methanol (CAS: 67-56-1, Mw = 32.04 g/mol, purity ≥ 99.8%), purchased from Pol-Aura (Morąg, Poland), was used as an auxiliary solvent for dilution and spectrophotometric analysis.

All chemicals were used as received without further purification.

### 3.2. Solubility Measurements

The equilibrium solubility of lidocaine and benzocaine in the prepared deep eutectic solvents (DESs) was determined using the isothermal shake-flask method.

DESs were prepared by combining a hydrogen bond acceptor (HBA: choline chloride or DL-menthol) with a hydrogen bond donor (HBD: tetraethylene glycol, triethylene glycol, diethylene glycol, ethylene glycol, 1,2-propanediol, and 1,3-butanediol) in molar ratios of 1:2, 1:3, and 1:4. The mixtures were heated and stirred until a clear, homogeneous liquid was formed. Subsequently, an excess amount of the respective active pharmaceutical ingredient (API) was added to each DES to ensure the formation of a saturated system.

Nominally ‘dry’ DES were prepared in closed vessels with minimal air exposure to minimize hygroscopic water uptake (typically resulting in <1 wt% residual water [55,69,70]). This approach follows standard laboratory practice in DES solubility studies and ensures that the distinction between dry and intentionally hydrated systems remains meaningful.

The prepared API-DES mixtures were placed in a thermostated water bath and incubated at a constant temperature of 25 °C for 24 h in an Orbital Shaker Incubator ES-20/60 from Biosan (Riga, Latvia). Continuous agitation at 60 rev/min was applied to reach thermodynamic equilibrium. After incubation, the saturated solutions were separated from the excess solid phase by filtration using 0.22 μm syringe filters. The filtered samples were then accurately weighed, diluted with methanol, and analyzed spectrophotometrically using an A360 spectrophotometer from AOE Instruments (Shanghai, China). All experiments were performed in triplicate, and the solubility was calculated and reported as the mean value with the corresponding standard deviation.

The solubility determination was based on initially prepared calibration curves. Stock solutions of both APIs were prepared by dissolving precisely weighed amounts of lidocaine and benzocaine in methanol. A series of working standard solutions was then obtained through serial dilution to establish calibration curves. The UV-Vis spectra of the solutions were recorded in the 190–500 nm wavelength range with 1 nm resolution using the A360 spectrophotometer. The absorption maxima were identified as λ_max_ = 263 nm for lidocaine and λ_max_ = 286 nm for benzocaine. The absorbance values recorded at these wavelengths were used to obtain the calibration curves, as well as solubility measurements. Three separate calibration curves were prepared for each API and the final curves were obtained via averaging the results.

The linear regression equations of the final calibration curves were found as A = 1.4853 ∙ C—0.0031 for lidocaine and A = 123.8788 ∙ C—0.0201 for benzocaine (A denoting absorbance values and C denoting concentration expressed in mg/mL), with corresponding determination coefficients equal R^2^ = 0.9994 and R^2^ = 0.9992 for lidocaine and benzocaine, respectively. The calibration curves were validated by calculating the limits of detection (LOD) and quantification (LOQ) based on the standard deviation of the residuals and the slope of the regression lines. For lidocaine these parameters were calculated as LOD = 0.0173 mg/mL and LOQ = 0.0524 mg/mL, while for benzocaine as LOD = 0.0004 mg/mL and LOQ = 0.0012 mg/mL.

### 3.3. Experimental Dataset Composition

The compiled dataset used for PCA and clustering was assembled from previously published API-DES solubility datasets covering ibuprofen and ketoprofen [71], ferulic acid [72], curcumin [73], caffeine [74], theobromine [75], theophylline [76], sulfacetamide and sulfanilamide [77], probenecid, sulfamethazine, sulfamethoxazole, and sulfasalazine [78], dapsone [79], edaravone [80], flufenamic acid [26], syringic, p-coumaric, and caffeic acids [32], as well as mefenamic and niflumic acids [81], and was supplemented with the new experimental data reported here for lidocaine and benzocaine. The descriptor generation and PCA/K-means workflow followed the approach described in our previous classification study [29]. The compiled solubility dataset used for the PCA and clustering comprised data from 21 APIs and included N = 2266 dry DES and N = 1051 wet DES datapoints. The new experimental data for benzocaine and lidocaine (2 additional APIs) were used exclusively for out-of-sample projection and validation. Dry DES systems correspond to those reported as anhydrous (x_w_ = 0 or negligible moisture as per source), while wet/aqueous systems include those with explicitly added water (x_w_ > 0). Compositions were incorporated directly into COSMO-RS calculations without further normalization, preserving the heterogeneous hydration levels reported in the original studies. This is consistent with the protocol in our previous classification study [29].

### 3.4. Computational Descriptor Generation

All molecular descriptors were derived analogically to our previous study [29] from first-principles calculations using the COSMO-RS (Conductor-like Screening Model for Real Solvents) methodology [82,83,84]. There are three sequential stages of the protocol toward descriptor collection. It starts with conformational analysis, which included comprehensive conformer sampling for each solute and solvent component using COSMOconf [85] with the default protocol. Up to ten lowest-energy conformers were retained for subsequent thermodynamic calculations to ensure representative sampling of conformational space. Geometry optimization and COSMO file generation were performed in TURBOMOLE [86] at the RI-BP/TZVP level with TZVPD-FINE basis set parameterization (BP_TZVPD_FINE_24.ctd), following established protocols [87,88,89]. Then the required cosmo and energy files for COSMOtherm [90] to compute interaction energies and chemical potentials were gathered for each API-DES combination. The descriptors enumerated in Table 1 were extracted from the last mixture calculation provided by output files. Notably, full solid–liquid equilibrium calculations were performed using experimentally determined fusion parameters. Melting temperatures (T_m_) and enthalpies of fusion (ΔH_fus_) were compiled as averaged literature values [91], with the entropy of fusion approximated as ΔS_fus_ ≈ ΔH_fus_/T_m_ and the Gibbs free energy of fusion calculated as ΔG_fus_ = ΔH_fus_ − TΔS_fus_. These values were provided in supporting materials along with the whole descriptors collection.

## 4. Conclusions

This study presents a data-driven taxonomy of solute–solvent systems in deep eutectic solvents (DES), based on COSMO-RS-derived descriptors and multivariate statistical analysis. Principal component analysis revealed that the complex descriptor space can be reduced to a small number of physically interpretable axes corresponding to solvation driving force, API thermodynamic stability, solvent interaction profile, hydrogen-bond network strength, and hydration competition. Clustering in the reduced PCA space demonstrated that DES systems form a quasi-continuous thermodynamic landscape rather than a set of sharply separated classes. Nevertheless, discretization of this space enabled identification of five distinct solvation regimes, each corresponding to different balances of intermolecular interactions and thermodynamic constraints. Two dominant solubilization mechanisms were identified: interaction-driven solvation, associated with strong API–DES affinity and high solubility, and destabilization-driven solvation, characterized by reduced API stability but lower overall efficiency. The presence of water was shown to play a critical role as a thermodynamic structuring agent, reducing the effective dimensionality of the system and promoting the emergence of more distinct solvation regimes. Rather than introducing new behavior, water redistributes systems within the existing thermodynamic space, selectively favoring regions associated with improved solubility. The developed framework was validated through projection of selected APIs (benzocaine and lidocaine), which exhibited distinct and interpretable distributions within the identified regimes. These results confirm that the proposed taxonomy captures meaningful physicochemical behavior and can be used to rationalize API-specific solubility trends.

In conclusion, it is worth adding that this work demonstrates that combining COSMO-RS descriptors with data-driven analysis enables construction of a physically grounded and predictive framework for DES-based solubilization. The approach provides a foundation for mechanism-guided formulation design, offering a pathway toward more efficient and rational development of pharmaceutical systems.

## Figures and Tables

**Table 1:** Detailed explanations of the descriptors used in the study.

Descriptor	Acronym	Explanation
log(x^est^)	log(x)SLE	COSMO-RS derived solubility collected as decadal logarithm of mole fraction
$\mu_{API}^{o}$	mu_self	chemical potential of pure API
$\mu_{API}^{sat}$	mu_sat	chemical potential of API under saturated conditions
$\mu_{API}^{water}$	mu_water	chemical potential of API in water
$E_{API}^{tot}$	solute_Etot	total API interaction energy under saturated conditions
$E_{API}^{misfit}$	solute_Emisfit	electrostatic (misfit) contribution to API energy under saturated conditions
$E_{API}^{HB}$	solute_Ehbond	hydrogen-bonding contribution to API intermolecular interaction energies under saturated conditions
$E_{API}^{VdW}$	solute_Evdw	non-bonding contribution to API intermolecular interaction energies under saturated conditions
ln($\gamma_{API}$)	solute_gamma	natural logarithm of API activity coefficient under saturated conditions
$E_{DES}^{tot}$	solv_Etot	total interaction energies of DES ^1^ $E_{DES}^{tot}=\&\sum_{i=1}^{N=3(4)}x_{i}^{*}\cdot E_{i}^{tot}\cdot$
$E_{DES}^{Misfit}$	solv_Emisfit	electrostatic interaction energies of DES ^1^ $E_{DES}^{Misfit}=\sum_{i=1}^{N=3(4)}x_{i}^{*}\cdot E_{i}^{Misfit}\cdot$
$E_{DES}^{HB}$	solv_Ehbond	hydrogen bonding interaction energies of DES ^1^ $E_{DES}^{HB}=\sum_{i=1}^{N=3(4)}x_{i}^{*}\cdot E_{i}^{HB}\cdot$
$E_{DES}^{vdW}$	solv_Evdw	non-bonding interaction energies of DES ^1^ $E_{DES}^{vdW}=\sum_{i=1}^{N=3(4)}x_{i}^{*}\cdot E_{i}^{vdW}\cdot$
$\mu_{DES}^{sat}$	solv_mu	chemical potential of DES under saturated conditions ^1^ $\mu_{DES}^{sat}=\sum_{i=1}^{N=3(4)}x_{i}^{*}\cdot \mu_{i}\cdot$
$d\mu^{sat}$	rel_mu	relative value of chemical potential (μ): $d\mu^{sat}=\mu_{API}^{sat}-\mu_{DES}^{sat}$
$d\mu_{API}$	rel_mu_water	relative value of chemical potentials of API in saturated system with respect of water: $d\mu_{API}=\mu_{API}^{sat}-\mu_{API}^{water}$
${dE}_{DES}^{tot}$	rel_Etot	relative values of total interaction energy in the saturated system ${dE}_{DES}^{tot}=E_{API}^{tot}-E_{DES}^{tot}$
${dE}_{DES}^{Misfit}$	rel_Emisfit	relative value of the electrostatic contribution to intermolecular interaction energies: $dE_{DES}^{Misfit}=E_{API}^{Misfit}-E_{DES}^{Misfit}$
$dE_{DES}^{HB}$	rel_Ehbond	relative value of the hydrogen bonding contribution to intermolecular interaction energies $dE_{DES}^{HB}=E_{API}^{HB}-E_{DES}^{HB}$
${dE}_{DES}^{vdW}$	rel_Evdw	relative value of the non-bonding contribution to intermolecular interaction energies: $dE_{DES}^{vdW}=E_{API}^{vdW}-E_{DES}^{vdW}$

^1^ N = 3(4) refers to the number of the individual components: N = 3 in the case of dry DES systems (HBA, HBD, API) and N = 4 in the case of wet DES systems (addition of water).

## Data Availability

The original contributions presented in this study are included in the article/Supplementary Materials. Further inquiries can be directed to the corresponding author.

## Supplementary Materials

The following supporting information can be downloaded at https://www.mdpi.com/article/10.3390/molecules31091482/s1: S1. Solubility values of lidocaine and benzocaine in the studied DES systems.; Table S1.1. Solubility of lidocaine, expressed as its mass concentration (C_lidocaine_, mg/ml) and mole fraction (x_lidocaine_), in neat DES systems comprising choline chloride as HBA.; Table S1.2. Solubility of lidocaine, expressed as its mass concentration (C_lidocaine_, mg/ml) and mole fraction (x_lidocaine_), in neat DES systems comprising menthol as HBA.; Table S1.3. Solubility of lidocaine, expressed as its mass concentration (C_lidocaine_, mg/ml) and mole fraction (x_lidocaine_), in aqueous DES systems comprising choline chloride as HBA and 1,3-butanediol as HBD in a 1:3 molar ratio and varying water content (mole fraction, x_w_).; Table S1.4. Solubility of benzocaine, expressed as its mass concentration (C_benzocaine_, mg/ml) and mole fraction (x_benzocaine_), in neat DES systems comprising choline chloride as HBA.; Table S1.5. Solubility of benzocaine, expressed as its mass concentration (C_benzocaine_, mg/ml) and mole fraction (x_benzocaine_), in neat DES systems comprising menthol as HBA.; Table S1.6. Solubility of benzocaine, expressed as its mass concentration (C_benzocaine_, mg/ml) and mole fraction (x_benzocaine_), in aqueous DES systems comprising choline chloride as HBA and tetraethylene glycol as HBD in a 1:3 molar ratio and varying water content (mole fraction, x_w_).; S2. PC loading persistence.; Figure S2.1. Loading persistence analysis of PC1 characterizing the whole dataset.; Figure S2.2. Loading persistence analysis of PC2 characterizing the whole dataset.; Figure S2.3. Loading persistence analysis of PC3 characterizing the whole dataset.; Figure S2.4. Loading persistence analysis of PC4 characterizing the whole dataset.; Figure S2.5. Loading persistence analysis of PC5 characterizing the whole dataset.; S3. Loading values of dry and wet API-DES systems.; Table S3.1. All API-DES systems.; Table S3.2. Only non-water containing API-DES systems.; S4. Supplementary projections of the PCA space.; Figure S4.1. Projection of DES systems onto the PC1–PC3 plane, illustrating the relationship between solvation driving force (PC1) and DES interaction profile (PC3), which reflects the balance between dispersion and polarity-driven interactions.; Figure S4.2. Projection onto the PC1–PC4 plane, highlighting the role of DES hydrogen-bond network strength (PC4) in modulating solubility.; Figure S4.3. Projection onto the PC1–PC5 plane, showing the influence of hydration competition on solubility behavior. PC5 represents the relative affinity of the API for aqueous versus DES environments.
