# Supplementary material for: Navigating the Solubility Landscape of APIs in Deep Eutectic Solvents: A Data-Driven Thermodynamic Taxonomy of Solvation Regimes and Mechanisms
Source: Molecules. 2026 Apr 29;31(9):1482. doi: 10.3390/molecules31091482 (PMC13165394; doi:10.3390/molecules31091482)
Supplement: Supplementary file 1 [file molecules-31-01482-s001.zip › molecules-4255508-supplementary.pdf]

Supplementary materials

S1. Solubility values of lidocaine and benzocaine in the studied DES systems. 3

Table S1.1. Solubility of lidocaine, expressed as its mass concentration ($C_{lidocaine}$, mg/ml) and mole fraction ($x_{lidocaine}$), in neat DES systems comprising choline chloride as HBA. Solubilities are presented with standard deviation values in parentheses. 3

Table S1.2. Solubility of lidocaine, expressed as its mass concentration ($C_{lidocaine}$, mg/ml) and mole fraction ($x_{lidocaine}$), in neat DES systems comprising menthol as HBA. Solubilities are presented with standard deviation values in parentheses. 3

Table S1.3. Solubility of lidocaine, expressed as its mass concentration ($C_{lidocaine}$, mg/ml) and mole fraction ($x_{lidocaine}$), in aqueous DES systems comprising choline chloride as HBA and 1,3-butanediol as HBD in a 1:3 molar ratio and varying water content (mole fraction, x_w). Solubilities are presented with standard deviation values in parentheses. 4

Table S1.4. Solubility of benzocaine, expressed as its mass concentration ($C_{benzocaine}$, mg/ml) and mole fraction ($x_{benzocaine}$), in neat DES systems comprising choline chloride as HBA. Solubilities are presented with standard deviation values in parentheses. 4

Table S1.5. Solubility of benzocaine, expressed as its mass concentration ($C_{benzocaine}$, mg/ml) and mole fraction ($x_{benzocaine}$), in neat DES systems comprising menthol as HBA. Solubilities are presented with standard deviation values in parentheses. 5

Table S1.6. Solubility of benzocaine, expressed as its mass concentration ($C_{benzocaine}$, mg/ml) and mole fraction ($x_{benzocaine}$), in aqueous DES systems comprising choline chloride as HBA and tetraethylene glycol as HBD in a 1:3 molar ratio and varying water content (mole fraction, x_w). Solubilities are presented with standard deviation values in parentheses. 5

S2. PC loading persistence. 6

Figure S2.1. Loading persistence analysis of PC1 characterizing the whole dataset. 6

Figure S2.2. Loading persistence analysis of PC2 characterizing the whole dataset. 6

Figure S2.3. Loading persistence analysis of PC3 characterizing the whole dataset. 7

Figure S2.4. Loading persistence analysis of PC4 characterizing the whole dataset. 7

Figure S2.5. Loading persistence analysis of PC5 characterizing the whole dataset. 8

S3. Loading values of dry and wet API-DES systems. 9

Table S3.1. All API-DES systems. 9

Table S3.2. Only non-water containing API-DES systems. 10

S4. Supplementary projections of the PCA space. 11

Figure S4.1. Projection of DES systems onto the PC1–PC3 plane, illustrating the relationship between solvation driving force (PC1) and DES interaction profile (PC3), which reflects the balance between dispersion and polarity-driven interactions. While solubility trends remain primarily governed by PC1, separation along PC3 reveals differentiation between dispersion-dominated and electrostatic-dominated solvent environments. The distinction between clusters C1 and C4 becomes more apparent, indicating that systems with similar solvation driving forces may differ in their underlying interaction mechanisms. 11

Figure S4.2. Projection onto the PC1–PC4 plane, highlighting the role of DES hydrogen-bond network strength (PC4) in modulating solubility. While PC1 governs the primary solvation driving force, variation along PC4 reveals differences in solvent structural cohesion. The interaction-driven regime (C4) is associated with more defined hydrogen-bond network characteristics, whereas the

destabilization-driven regime (C1) spans a broader range of solvent structuring. Systems with weak hydrogen-bond networks (low PC4), particularly in cluster C2, exhibit reduced solubility despite favorable interaction energies. 12

Figure S4.3. Projection onto the PC1–PC5 plane, showing the influence of hydration competition on solubility behavior. PC5 represents the relative affinity of the API for aqueous versus DES environments. While clustering remains primarily defined by PC1, variation along PC5 reveals secondary differentiation within clusters, particularly between C1 and C4. Systems with higher PC5 values exhibit stronger preference for DES over water, whereas lower values indicate increased hydration tendency, which may limit solubility enhancement in DES. 13

S1. Solubility values of lidocaine and benzocaine in the studied DES systems.

Table S1.1. Solubility of lidocaine, expressed as its mass concentration ($C_{lidocaine}$, mg/ml) and mole fraction ($x_{lidocaine}$), in neat DES systems comprising choline chloride as HBA. Solubilities are presented with standard deviation values in parentheses.

**Hydrogen Bond Donor**	**HBA:HBD ratio**	**$C_{lidocaine}$ [mg/ml]**	**$x_{lidocaine}$**
1,3-butanediol	1:3	68.477 (±0.597)	0.0298 (±0.0002)
1,3-butanediol	1:2	58.685 (±0.535)	0.0263 (±0.0002)
1,3-butanediol	1:4	52.941 (±0.697)	0.0225 (±0.0004)
1,2-propanediol	1:3	57.204 (±0.597)	0.0220 (±0.0002)
1,2-propanediol	1:2	55.145 (±0.410)	0.0222 (±0.0002)
1,2-propanediol	1:4	50.917 (±0.705)	0.0189 (±0.0002)
tetraethylene glycol	1:3	47.934 (±0.464)	0.0333 (±0.0005)
tetraethylene glycol	1:2	45.578 (±0.363)	0.0313 (±0.0002)
tetraethylene glycol	1:4	41.172 (±0.454)	0.0290 (±0.0003)
triethylene glycol	1:3	47.510 (±0.394)	0.0272 (±0.0001)
triethylene glycol	1:2	42.585 (±0.356)	0.0244 (±0.0002)
triethylene glycol	1:4	39.605 (±0.343)	0.0226 (±0.0002)
diethylene glycol	1:3	41.740 (±0.389)	0.0186 (±0.0002)
diethylene glycol	1:2	38.502 (±0.371)	0.0176 (±0.0002)
diethylene glycol	1:4	32.239 (±0.297)	0.0140 (±0.0001)
ethylene glycol	1:3	37.201 (±0.185)	0.0118 (±0.0001)
ethylene glycol	1:2	35.533 (±0.324)	0.0121 (±0.0001)
ethylene glycol	1:4	30.401 (±0.318)	0.0092 (±0.0001)

Table S1.2. Solubility of lidocaine, expressed as its mass concentration ($C_{lidocaine}$, mg/ml) and mole fraction ($x_{lidocaine}$), in neat DES systems comprising menthol as HBA. Solubilities are presented with standard deviation values in parentheses.

**Hydrogen Bond Donor**	**HBA:HBD ratio**	**$C_{lidocaine}$ [mg/ml]**	**$x_{lidocaine}$**
1,3-butanediol	1:3	327.213 (±3.266)	0.1844 (±0.0025)
1,3-butanediol	1:2	291.189 (±2.678)	0.1702 (±0.0018)
1,3-butanediol	1:4	259.518 (±1.947)	0.1327 (±0.0015)
1,2-propanediol	1:3	274.661 (±3.013)	0.1317 (±0.0020)
1,2-propanediol	1:2	239.527 (±2.594)	0.1229 (±0.0014)
1,2-propanediol	1:4	201.127 (±0.907)	0.0875 (±0.0008)
tetraethylene glycol	1:3	152.632 (±1.947)	0.1151 (±0.0021)
tetraethylene glycol	1:2	139.073 (±3.467)	0.1059 (±0.0025)
tetraethylene glycol	1:4	120.962 (±2.228)	0.0901 (±0.0013)
triethylene glycol	1:3	145.270 (±1.425)	0.0926 (±0.0008)
triethylene glycol	1:2	141.693 (±1.886)	0.0927 (±0.0015)
triethylene glycol	1:4	104.263 (±0.857)	0.0643 (±0.0007)
diethylene glycol	1:3	144.647 (±1.089)	0.0738 (±0.0007)
diethylene glycol	1:2	112.869 (±1.558)	0.0603 (±0.0008)
diethylene glycol	1:4	100.479 (±1.273)	0.0482 (±0.0007)
ethylene glycol	1:3	122.148 (±1.648)	0.0457 (±0.0007)
ethylene glycol	1:2	103.900 (±1.578)	0.0429 (±0.0007)
ethylene glycol	1:4	97.731 (±1.103)	0.0335 (±0.0004)

Table S1.3. Solubility of lidocaine, expressed as its mass concentration ($C_{lidocaine}$, mg/ml) and mole fraction ($x_{lidocaine}$), in aqueous DES systems comprising choline chloride as HBA and 1,3-butanediol as HBD in a 1:3 molar ratio and varying water content (mole fraction, x_w). Solubilities are presented with standard deviation values in parentheses.

x_w	$C_{lidocaine}$ [mg/ml]	$x_{lidocaine}$
0.0	68.477 (±0.597)	0.0298 (±0.0002)
0.1	78.486 (±0.976)	0.0316 (±0.0004)
0.2	65.767 (±0.381)	0.0240 (±0.0001)
0.3	52.399 (±0.226)	0.0172 (±0.0002)
0.4	39.481 (±0.392)	0.0115 (±0.0001)
0.5	28.336 (±0.382)	0.0072 (±0.0002)
0.6	19.042 (±0.142)	0.0041 (±0.0001)
0.7	12.168 (±0.130)	0.0022 (±0.0001)
0.8	8.021 (±0.091)	0.0012 (±0.0001)
0.9	5.802 (±0.079)	0.0006 (±0.0001)

Table S1.4. Solubility of benzocaine, expressed as its mass concentration ($C_{benzocaine}$, mg/ml) and mole fraction ($x_{benzocaine}$), in neat DES systems comprising choline chloride as HBA. Solubilities are presented with standard deviation values in parentheses.

Hydrogen Bond Donor	HBA:HBD ratio	$C_{benzocaine}$ [mg/ml]	$x_{benzocaine}$
1,3-butanediol	1:3	63.652 (±1.337)	0.0388 (±0.0007)
1,3-butanediol	1:2	52.208 (±0.811)	0.0326 (±0.0004)
1,3-butanediol	1:4	42.536 (±0.380)	0.0252 (±0.0003)
1,2-propanediol	1:3	61.015 (±1.803)	0.0330 (±0.0011)
1,2-propanediol	1:2	48.403 (±1.077)	0.0273 (±0.0006)
1,2-propanediol	1:4	39.403 (±0.981)	0.0205 (±0.0005)
tetraethylene glycol	1:3	67.827 (±0.625)	0.0655 (±0.0005)
tetraethylene glycol	1:2	56.999 (±0.937)	0.0546 (±0.0009)
tetraethylene glycol	1:4	46.050 (±0.503)	0.0454 (±0.0006)
triethylene glycol	1:3	54.768 (±2.233)	0.0441 (±0.0017)
triethylene glycol	1:2	45.548 (±0.954)	0.0366 (±0.0008)
triethylene glycol	1:4	33.702 (±0.442)	0.0270 (±0.0004)
diethylene glycol	1:3	35.912 (±0.934)	0.0225 (±0.0006)
diethylene glycol	1:2	31.188 (±0.954)	0.0200 (±0.0006)
diethylene glycol	1:4	27.180 (±0.783)	0.0166 (±0.0005)
ethylene glycol	1:3	29.333 (±0.625)	0.0130 (±0.0003)
ethylene glycol	1:2	26.019 (±0.329)	0.0124 (±0.0002)
ethylene glycol	1:4	22.998 (±0.349)	0.0098 (±0.0001)

Table S1.5. Solubility of benzocaine, expressed as its mass concentration ($C_{benzocaine}$, mg/ml) and mole fraction ($x_{benzocaine}$), in neat DES systems comprising menthol as HBA. Solubilities are presented with standard deviation values in parentheses.

**Hydrogen Bond Donor**	**HBA:HBD ratio**	**$C_{benzocaine}$ [mg/ml]**	**$x_{benzocaine}$**
1,3-butanediol	1:3	137.560 (±2.231)	0.0949 (±0.0016)
1,3-butanediol	1:2	116.157 (±3.764)	0.0842 (±0.0032)
1,3-butanediol	1:4	110.747 (±1.997)	0.0719 (±0.0014)
1,2-propanediol	1:3	119.473 (±2.865)	0.0701 (±0.0054)
1,2-propanediol	1:2	106.943 (±4.667)	0.0703 (±0.0033)
1,2-propanediol	1:4	99.057 (±1.383)	0.0563 (±0.0009)
tetraethylene glycol	1:3	126.362 (±4.451)	0.1292 (±0.0041)
tetraethylene glycol	1:2	111.596 (±2.098)	0.1158 (±0.0020)
tetraethylene glycol	1:4	100.180 (±0.922)	0.1022 (±0.0009)
triethylene glycol	1:3	104.910 (±1.308)	0.0911 (±0.0013)
triethylene glycol	1:2	97.177 (±1.352)	0.0863 (±0.0014)
triethylene glycol	1:4	93.030 (±0.783)	0.0791 (±0.0004)
diethylene glycol	1:3	103.501 (±0.933)	0.0717 (±0.0007)
diethylene glycol	1:2	95.743 (±0.750)	0.0701 (±0.0005)
diethylene glycol	1:4	88.639 (±1.099)	0.0586 (±0.0006)
ethylene glycol	1:3	90.999 (±0.717)	0.0467 (±0.0005)
ethylene glycol	1:2	84.341 (±3.578)	0.0479 (±0.0021)
ethylene glycol	1:4	80.618 (±1.065)	0.0383 (±0.0005)

Table S1.6. Solubility of benzocaine, expressed as its mass concentration ($C_{benzocaine}$, mg/ml) and mole fraction ($x_{benzocaine}$), in aqueous DES systems comprising choline chloride as HBA and tetraethylene glycol as HBD in a 1:3 molar ratio and varying water content (mole fraction, x_w). Solubilities are presented with standard deviation values in parentheses.

**x_w**	**$C_{benzocaine}$ [mg/ml]**	**$x_{benzocaine}$**
0.0	67.827 (±0.625)	0.0655 (±0.0005)
0.1	71.867 (±1.431)	0.0637 (±0.0013)
0.2	59.600 (±0.885)	0.0479 (±0.0007)
0.3	42.609 (±1.435)	0.0306 (±0.0011)
0.4	27.667 (±0.855)	0.0174 (±0.0006)
0.5	16.243 (±0.357)	0.0088 (±0.0002)
0.6	9.385 (±0.446)	0.0043 (±0.0002)
0.7	4.987 (±0.200)	0.0018 (±0.0001)
0.8	2.821 (±0.050)	0.0008 (±0.0001)
0.9	1.546 (±0.059)	0.0003 (±0.0001)

S2. PC loading persistence.

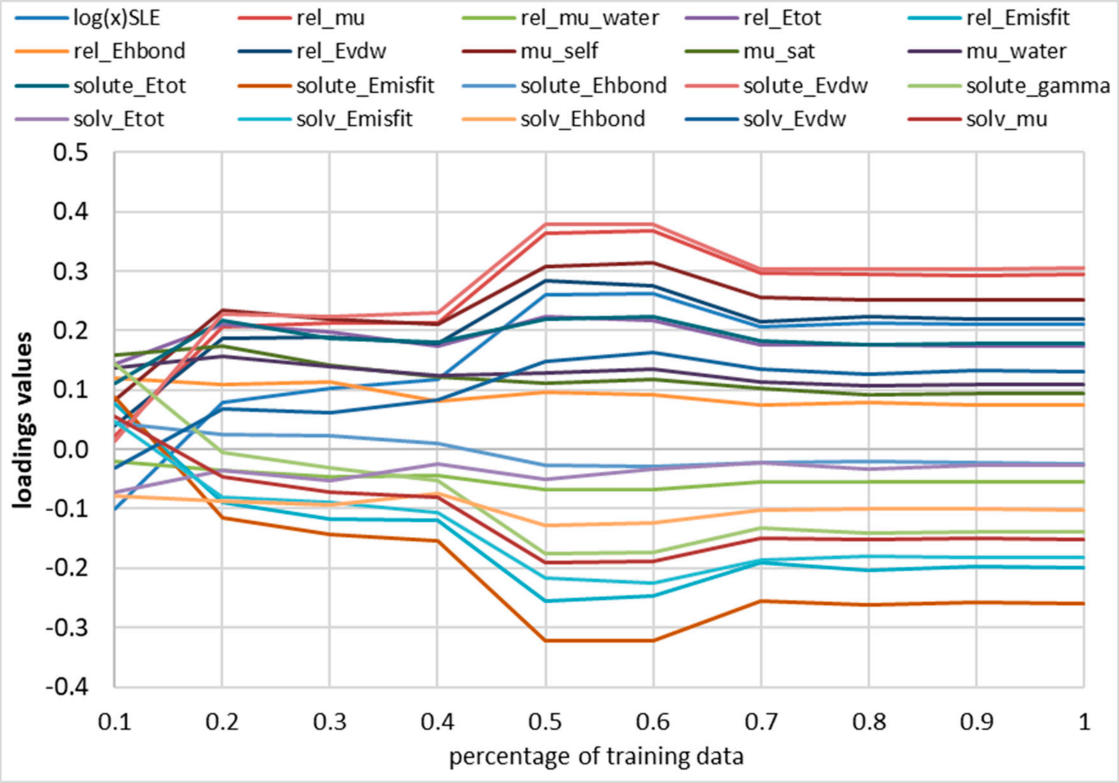

Figure S2.1. Loading persistence analysis of PC1 characterizing the whole dataset.

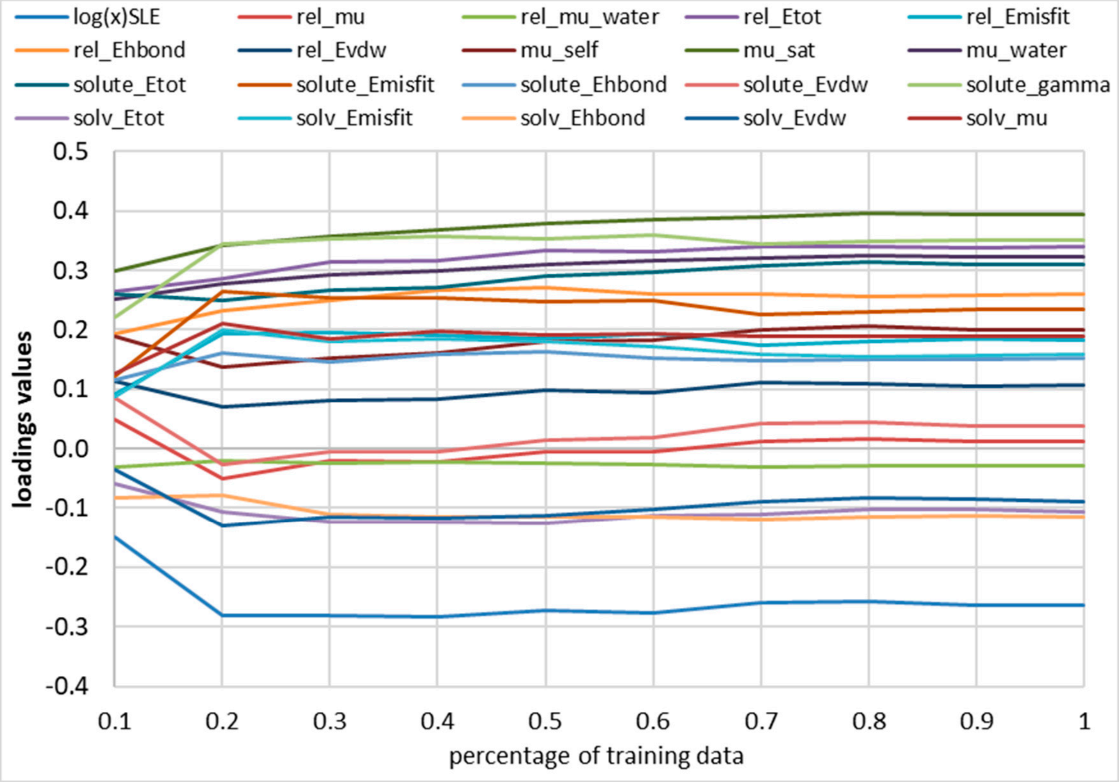

Figure S2.2. Loading persistence analysis of PC2 characterizing the whole dataset.

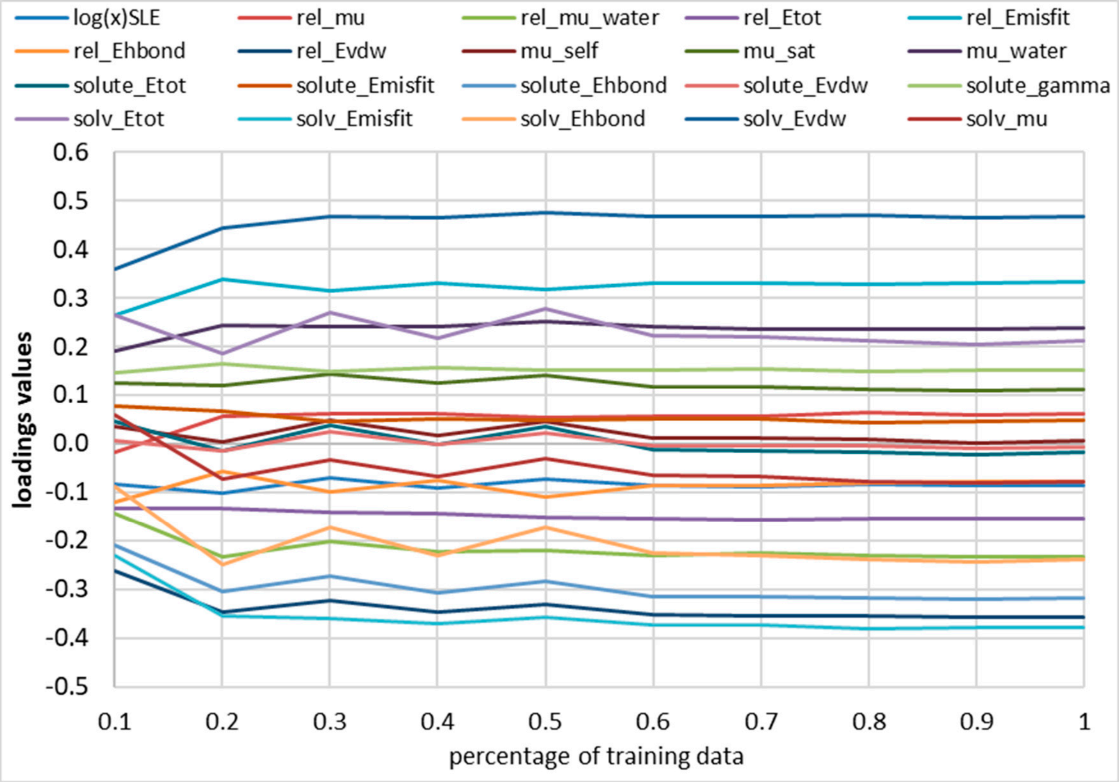

Figure S2.3. Loading persistence analysis of PC3 characterizing the whole dataset.

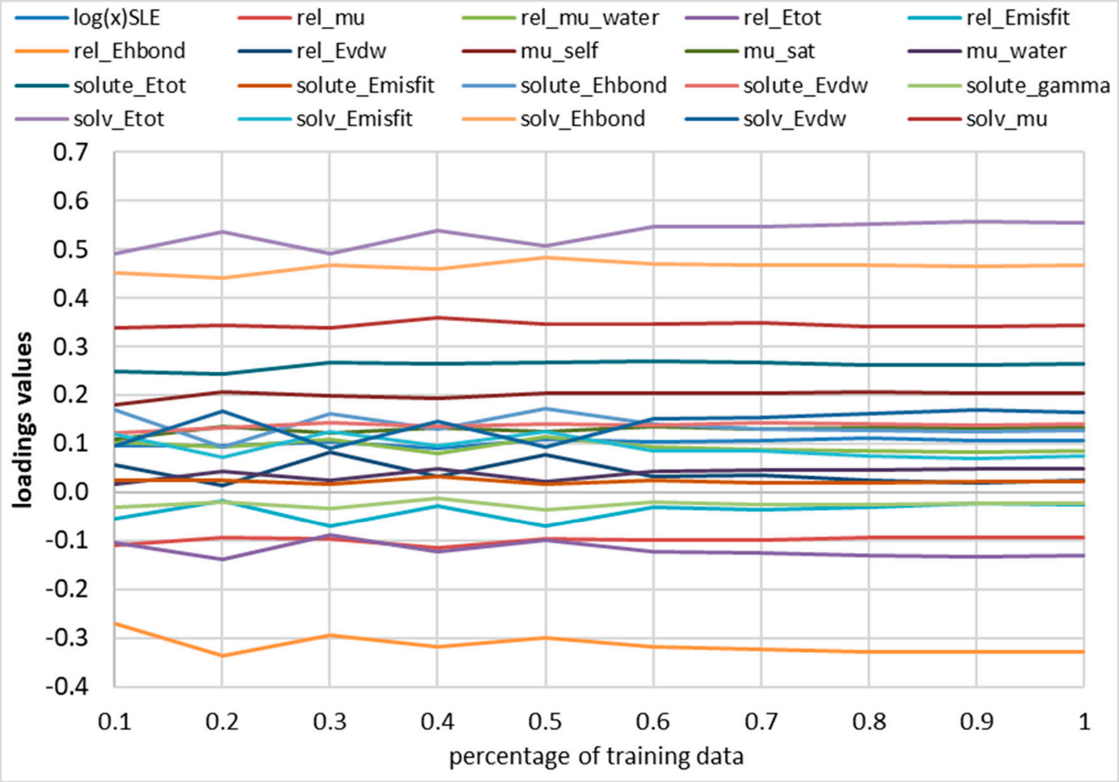

Figure S2.4. Loading persistence analysis of PC4 characterizing the whole dataset.

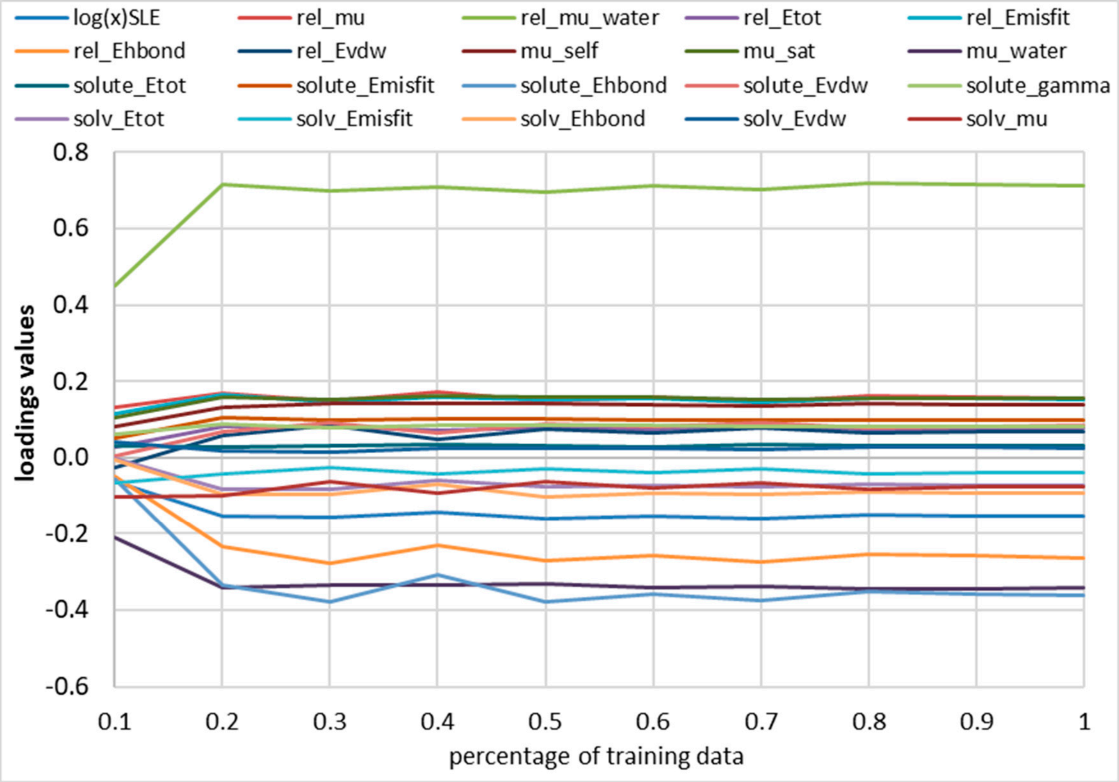

Figure S2.5. Loading persistence analysis of PC5 characterizing the whole dataset.

S3. Loading values of dry and wet API-DES systems.

Table S3.1. All API-DES systems.

	PC1	PC2	PC3	PC4	PC5
**solute_Evdw**	0.381	0.036	-0.007	0.141	0.084
**rel_mu**	0.367	0.011	0.061	-0.095	0.157
**solute_Emisfit**	-0.335	0.237	0.048	0.021	0.099
**mu_self**	0.306	0.200	0.006	0.205	0.139
**log(x)SLE**	0.274	-0.266	-0.086	0.105	-0.154
**rel_Evdw**	0.273	0.105	-0.357	0.024	0.069
**rel_Emisfit**	-0.259	0.186	0.332	-0.026	0.153
**solv_Emisfit**	-0.234	0.161	-0.379	0.074	-0.038
**solute_Etot**	0.211	0.311	-0.019	0.266	0.032
**rel_Etot**	0.205	0.340	-0.154	-0.131	0.076
**solv_mu**	-0.197	0.190	-0.079	0.343	-0.075
**solute_gamma**	-0.190	0.354	0.151	-0.022	0.082
**solv_Evdw**	0.167	-0.090	0.468	0.165	0.025
**mu_water**	0.122	0.324	0.237	0.049	-0.342
**solv_Ehbond**	-0.122	-0.115	-0.240	0.467	-0.094
**mu_sat**	0.101	0.396	0.112	0.134	0.156
**rel_Ehbond**	0.083	0.261	-0.079	-0.328	-0.264
**rel_mu_water**	-0.069	-0.028	-0.233	0.084	0.713
**solute_Ehbond**	-0.035	0.154	-0.319	0.127	-0.364
**solv_Etot**	-0.032	-0.106	0.210	0.555	-0.074
	**PC1**	**PC2**	**PC3**	**PC4**	**PC5**
**solute_Evdw**	Moderate	Weak	Weak	Weak	Weak
**rel_mu**	Moderate	Weak	Weak	Weak	Weak
**solute_Emisfit**	Moderate	Weak	Weak	Weak	Weak
**mu_self**	Weak	Weak	Weak	Weak	Weak
**log(x)SLE**	Weak	Weak	Weak	Weak	Weak
**rel_Evdw**	Weak	Weak	Moderate	Weak	Weak
**rel_Emisfit**	Weak	Weak	Moderate	Weak	Weak
**solv_Emisfit**	Weak	Weak	Moderate	Weak	Weak
**solute_Etot**	Weak	Weak	Weak	Weak	Weak
**rel_Etot**	Weak	Moderate	Weak	Weak	Weak
**solv_mu**	Weak	Weak	Weak	Moderate	Weak
**solute_gamma**	Weak	Moderate	Weak	Weak	Weak
**solv_Evdw**	Weak	Weak	Strong	Weak	Weak
**mu_water**	Weak	Moderate	Weak	Weak	Moderate
**solv_Ehbond**	Weak	Weak	Weak	Strong	Weak
**mu_sat**	Weak	Moderate	Weak	Weak	Weak
**rel_Ehbond**	Weak	Weak	Weak	Moderate	Weak
**rel_mu_water**	Weak	Weak	Weak	Weak	Strong
**solute_Ehbond**	Weak	Weak	Moderate	Weak	Moderate
**solv_Etot**	Weak	Weak	Weak	Strong	Weak

Table S3.2. Only non-water containing API-DES systems.

	PC1	PC2	PC3	PC4	PC5
**solute_Emisfit**	0.385	0.028	0.054	0.113	0.105
**log(x)SLE**	-0.370	-0.041	0.063	-0.039	-0.116
**solute_gamma**	0.344	0.151	0.035	0.165	0.057
**rel_Emisfit**	0.327	-0.047	0.057	0.385	0.069
**solute_Evdw**	-0.313	0.196	0.122	-0.094	0.108
**rel_mu**	-0.308	0.164	-0.051	0.295	0.060
**solv_Emisfit**	0.289	0.141	0.022	-0.398	0.113
**solv_mu**	0.251	0.062	0.270	-0.339	0.066
**rel_Evdw**	-0.209	0.281	-0.037	-0.330	0.144
**solv_Evdw**	-0.184	-0.150	0.279	0.415	-0.064
**mu_self**	-0.174	0.302	0.216	0.061	0.157
**mu_water**	0.138	0.360	0.071	0.186	-0.124
**mu_sat**	0.110	0.349	0.191	0.194	0.153
**rel_mu_water**	-0.081	-0.081	0.244	-0.015	0.607
**solute_Etot**	-0.049	0.345	0.268	0.066	-0.018
**rel_Etot**	-0.035	0.396	-0.125	0.000	0.102
**solute_Ehbond**	0.035	0.240	0.123	-0.105	-0.567
**rel_Ehbond**	0.016	0.272	-0.317	0.115	-0.233
**solv_Ehbond**	0.013	-0.083	0.460	-0.221	-0.253
**solv_Etot**	-0.011	-0.139	0.506	0.081	-0.167
	**PC1**	**PC2**	**PC3**	**PC4**	**PC5**
**solute_Evdw**	Moderate	Weak	Weak	Weak	Weak
**rel_mu**	Moderate	Weak	Weak	Weak	Weak
**solute_Emisfit**	Moderate	Weak	Weak	Weak	Weak
**mu_self**	Moderate	Weak	Weak	Moderate	Weak
**log(x)SLE**	Moderate	Weak	Weak	Weak	Weak
**rel_Evdw**	Weak	Weak	Weak	Weak	Weak
**rel_Emisfit**	Weak	Weak	Weak	Moderate	Weak
**solv_Emisfit**	Weak	Weak	Weak	Moderate	Weak
**solute_Etot**	Weak	Weak	Weak	Moderate	Weak
**rel_Etot**	Weak	Weak	Weak	Moderate	Weak
**solv_mu**	Weak	Weak	Weak	Weak	Weak
**solute_gamma**	Weak	Moderate	Weak	Weak	Weak
**solv_Evdw**	Weak	Moderate	Weak	Weak	Weak
**mu_water**	Weak	Weak	Weak	Weak	Strong
**solv_Ehbond**	Weak	Moderate	Weak	Weak	Weak
**mu_sat**	Weak	Moderate	Weak	Weak	Weak
**rel_Ehbond**	Weak	Weak	Weak	Weak	Strong
**rel_mu_water**	Weak	Weak	Moderate	Weak	Weak
**solute_Ehbond**	Weak	Weak	Strong	Weak	Weak
**solv_Etot**	Weak	Weak	Strong	Weak	Weak

S4. Supplementary projections of the PCA space.

Supplementary projections of the PCA space are provided to validate the robustness of clustering and to illustrate the contribution of higher-order physicochemical factors governing solubility.

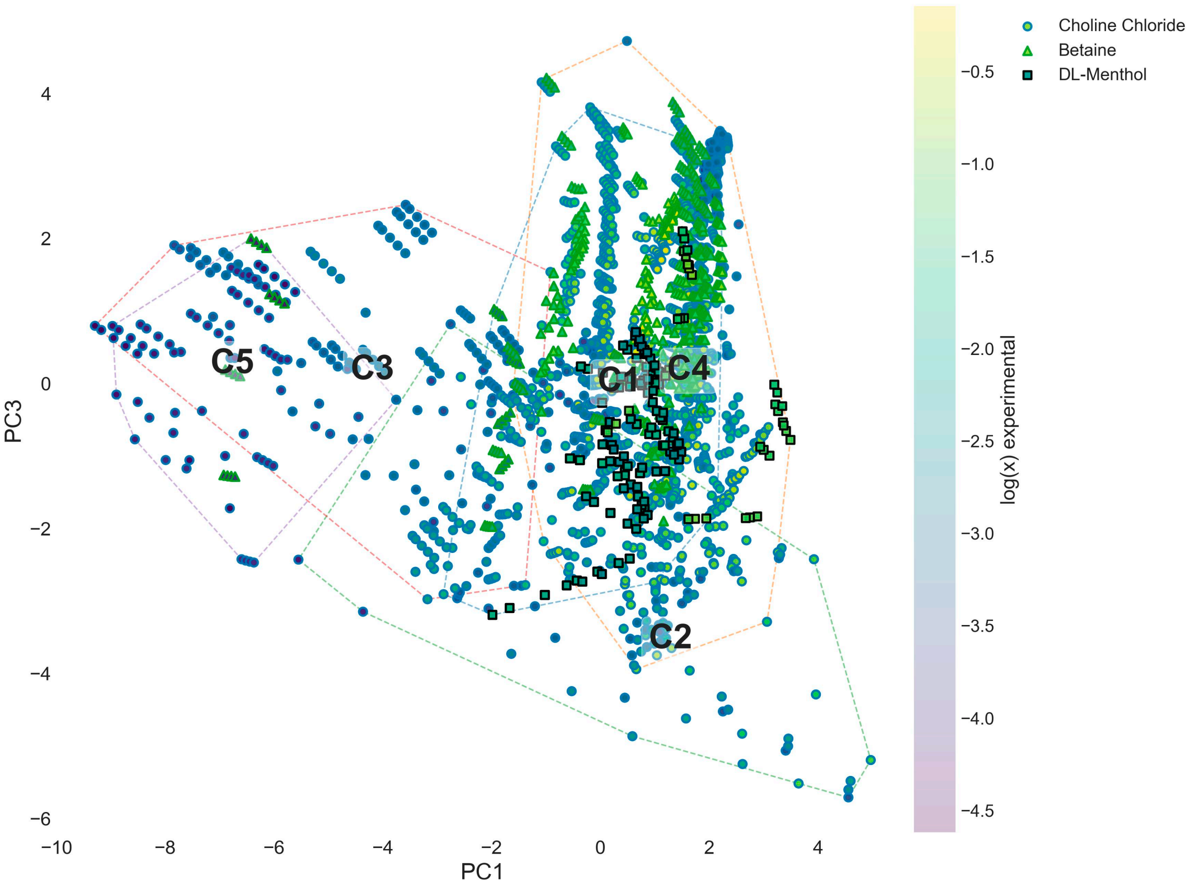

Figure S4.1. Projection of DES systems onto the PC1–PC3 plane, illustrating the relationship between solvation driving force (PC1) and DES interaction profile (PC3), which reflects the balance between dispersion and polarity-driven interactions. While solubility trends remain primarily governed by PC1, separation along PC3 reveals differentiation between dispersion-dominated and electrostatic-dominated solvent environments. The distinction between clusters C1 and C4 becomes more apparent, indicating that systems with similar solvation driving forces may differ in their underlying interaction mechanisms.

The projection of DES systems onto the PC1–PC3 plane (Figure S4.1) provides complementary insight into the role of solvent interaction characteristics beyond the primary solvation-driving axis. While PC1 retains its interpretation as the global solvation driving force, PC3 introduces discrimination based on the balance between dispersion and polarity within the solvent environment. A clear separation of clusters along PC1 is preserved, confirming that solubility trends remain primarily governed by the solvation driving force. However, additional structuring along PC3 reveals differentiation between dispersion-dominated and polarity-dominated DES systems. Clusters located at higher PC3 values correspond to systems with stronger dispersion contributions, while lower PC3 values indicate increasing influence of electrostatic and hydrogen-bonding interactions. Importantly, clusters C1 and C4, which partially overlap in the PC1–PC2 representation, become more distinguishable along PC3. This indicates that although these clusters may exhibit similar overall solvation driving forces, they differ in the underlying solvent interaction mechanisms. In particular,

the interaction-driven solvation regime (C4) is more tightly associated with specific solvent interaction profiles, whereas the destabilization-driven regime (C1) spans a broader range of solvent characteristics. The distribution of hydrogen bond acceptors further supports this interpretation. DL-menthol-based systems are preferentially located in regions corresponding to dispersion-dominated interactions, while choline chloride and betaine systems occupy a wider range of PC3 values, reflecting their broader interaction versatility. The presented PC1–PC3 projection confirms that clustering is not solely driven by solubility magnitude but also reflects distinct physicochemical interaction regimes. This supports the interpretation of DES systems as occupying a multidimensional thermodynamic space in which both interaction strength and interaction type govern solubility behavior.

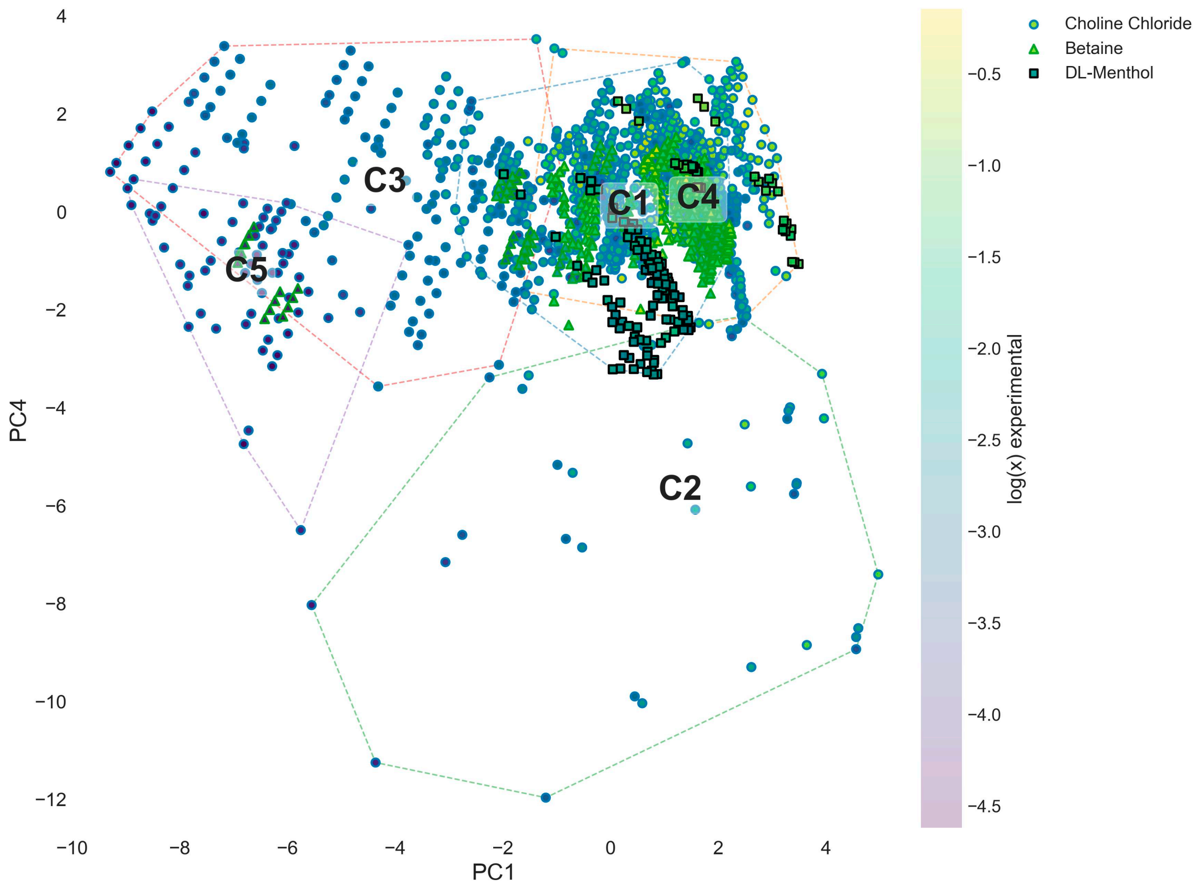

Figure S4.2. Projection onto the PC1–PC4 plane, highlighting the role of DES hydrogen-bond network strength (PC4) in modulating solubility. While PC1 governs the primary solvation driving force, variation along PC4 reveals differences in solvent structural cohesion. The interaction-driven regime (C4) is associated with more defined hydrogen-bond network characteristics, whereas the destabilization-driven regime (C1) spans a broader range of solvent structuring. Systems with weak hydrogen-bond networks (low PC4), particularly in cluster C2, exhibit reduced solubility despite favorable interaction energies.

The projection onto the PC1–PC4 plane (Figure S4.2) highlights the role of hydrogen-bond network strength in modulating solubility behavior within DES systems. While PC1 continues to represent the global solvation driving force, PC4 introduces differentiation based on the collective hydrogen-bonding interactions and structural cohesion of the solvent. A clear separation along PC1 is maintained, confirming that solubility trends are primarily governed by API–DES affinity. However, the distribution along PC4 reveals additional structuring, particularly within the high-solubility region. Clusters C1 and C4, which occupy similar ranges of PC1, exhibit distinct positions along PC4,

indicating that differences in hydrogen-bond network strength contribute to their separation. In particular, cluster C4 is associated with higher PC4 values, corresponding to more structured and cohesive hydrogen-bond networks, whereas cluster C1 spans a broader range of PC4, reflecting greater variability in solvent structuring. This suggests that optimal solvation (C4) is not only driven by favorable interaction strength but also supported by well-defined solvent organization. Cluster C2 is clearly distinguished by strongly negative PC4 values, indicating weak or disrupted hydrogen-bond networks. Despite occasionally favorable solvation driving forces, these systems exhibit poor solubility, highlighting the importance of solvent cohesion in enabling effective solute accommodation. The distribution of hydrogen bond acceptors further supports this interpretation. DL-menthol-based systems are predominantly located in regions of lower PC4 values, consistent with weaker hydrogen-bonding capabilities, whereas choline chloride and betaine systems extend toward higher PC4 values, reflecting their ability to form structured hydrogen-bond networks. Hence, the PC1–PC4 projection demonstrates that hydrogen-bond network strength acts as a secondary but critical factor in defining solvation regimes, complementing the primary solvation driving force captured by PC1.

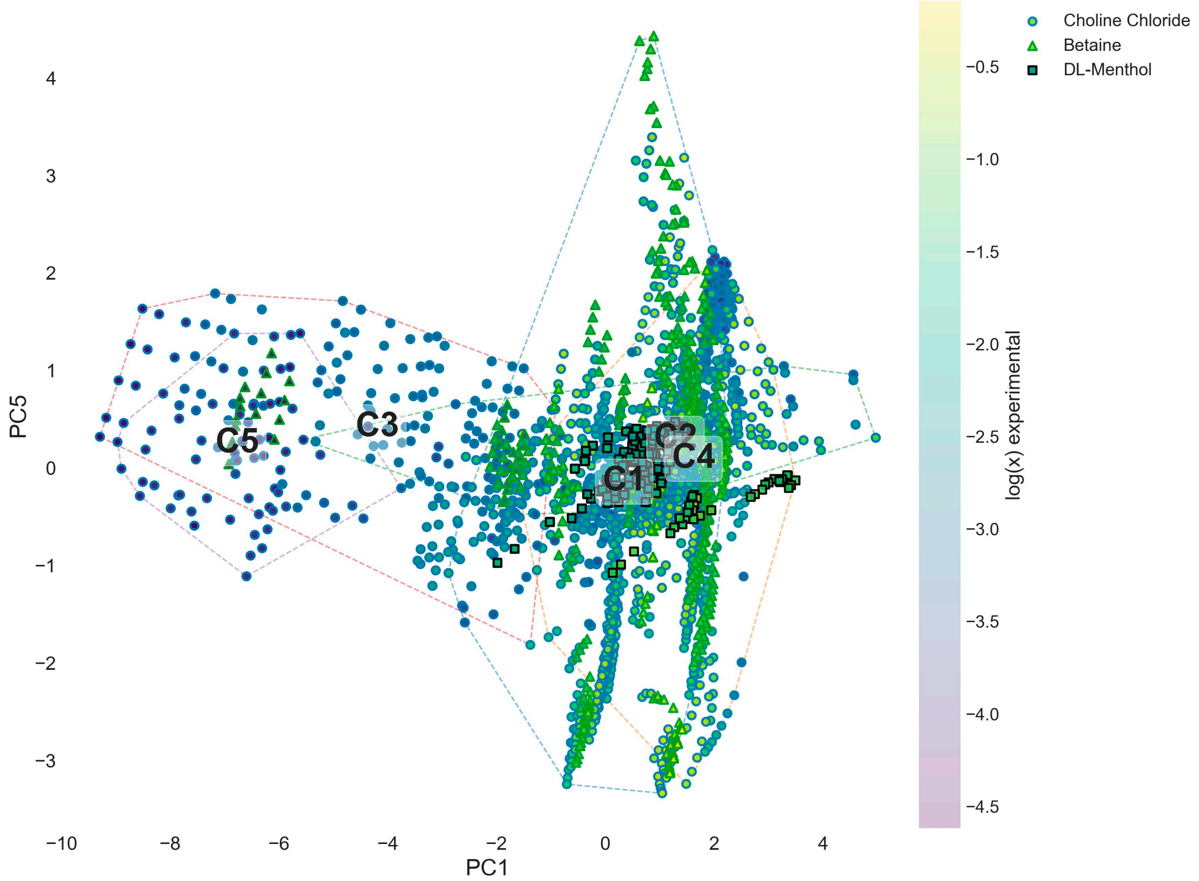

Figure S4.3. Projection onto the PC1–PC5 plane, showing the influence of hydration competition on solubility behavior. PC5 represents the relative affinity of the API for aqueous versus DES environments. While clustering remains primarily defined by PC1, variation along PC5 reveals secondary differentiation within clusters, particularly between C1 and C4. Systems with higher PC5 values exhibit stronger preference for DES over water, whereas lower values indicate increased hydration tendency, which may limit solubility enhancement in DES.

The projection onto the PC1–PC5 plane (Figure S4.3) highlights the influence of hydration effects and the competition between water and DES environments in governing solubility behavior. While PC1 retains its role as the primary solvation driving force, PC5 introduces an additional dimension related to the relative affinity of the API for aqueous versus DES phases. A clear

separation along PC1 is preserved, confirming that solubility trends remain primarily controlled by API–DES affinity. However, the spread of data along PC5 reveals significant variability associated with hydration effects, particularly within clusters occupying similar PC1 regions. This indicates that systems with comparable solvation driving forces may differ substantially in their preference for water versus DES environments. Clusters C1 and C4, which overlap in PC1, show partial differentiation along PC5, suggesting that hydration-related thermodynamic factors contribute to their distinction. In particular, systems with higher PC5 values correspond to greater preference for DES over water, while lower PC5 values indicate stronger affinity toward aqueous environments, potentially limiting solubility enhancement in DES. Cluster C5 remains well separated along PC1, confirming its characterization as a low-solubility regime, but also exhibits a relatively narrow distribution in PC5, indicating that poor solubility in these systems is primarily driven by unfavorable API–DES interactions rather than hydration competition. In contrast, clusters located in the central region (C1–C4) display broader distributions along PC5, reflecting the increasing importance of water-related effects in modulating solubility. The distribution of hydrogen bond acceptors further supports this interpretation. Systems based on choline chloride and betaine span a wide range of PC5 values, indicating variability in hydration sensitivity, whereas DL-menthol systems tend to occupy regions with lower PC5 variability, consistent with their reduced affinity for water and more hydrophobic character. The PC1–PC5 projection confirms that hydration effects act as an additional, secondary factor influencing solubility, particularly in systems where primary interaction-driven mechanisms are comparable. This supports the interpretation that water introduces an additional layer of thermodynamic competition, modulating but not dominating the primary solvation driving forces in DES systems.
